# PIK3CA Mutations Downregulate PPT1 to Promote Adipogenesis by Suppressing P300 Depalmitoylation and Phase Separation

**DOI:** 10.1002/advs.202523139

**Published:** 2026-01-29

**Authors:** Hongrui Chen, Zening Huang, Rui Chang, Wei Gao, Yajing Qiu, Bin Sun, Chen Hua, Xiaoxi Lin

**Affiliations:** ^1^ Department of Plastic & Reconstructive Surgery Shanghai Ninth People's Hospital Shanghai Jiao Tong University School of Medicine Shanghai China; ^2^ Department of Laser and Aesthetic Medicine Shanghai Ninth People's Hospital Shanghai Jiao Tong University School of Medicine Shanghai China; ^3^ Department of Gastric Surgery Fujian Medical University Union Hospital Fuzhou China

**Keywords:** adipogenesis, palmitoylation, phase separation, PIK3CA

## Abstract

*PIK3CA* mutations drive benign adipose overgrowth in facial infiltrating lipomatosis (FIL), but the downstream molecular mechanisms remain incompletely understood. This study investigated the role of palmitoyl‐protein thioesterase 1 (PPT1)‐mediated depalmitoylation in regulating aberrant adipogenesis induced by mutant *PIK3CA*. Using single‐cell RNA‐seq, molecular dynamics simulations, and functional assays in primary human FIL adipose‐derived stem and progenitor cells (ASPCs), immortalized cell lines, and mouse models, we dissected the signaling pathway linking *PIK3CA* mutation to adipogenesis. Techniques included ChIP‐qPCR, acyl‐biotin exchange assays, luciferase reporter assays, and RNA/ATAC sequencing. *PIK3CA* mutations transcriptionally repressed PPT1 via PI3K‐AKT‐c‐JUN signaling. Downregulated PPT1 enhanced palmitoylation of the transcriptional coactivator P300 at C1176. This modification stabilized P300 by impairing its interaction with HSC70 and subsequent chaperone‐mediated lysosomal degradation. Furthermore, C1176 palmitoylation inhibited P300 phase separation, thereby preserving its histone acetyltransferase activity. Sustained P300 activity promoted chromatin accessibility and expression of adipogenic genes, driving excessive adipogenesis in FIL. These findings established a novel “palmitoylation‐phase separation‐epigenetic regulation” axis in cellular fate determination and revealed PPT1 and P300 as potential therapeutic targets for FIL.

## Graphical abstract

This study demonstrates that somatic PIK3CA mutations suppress PPT1 expression via activation of the PI3K–AKT–c‐JUN axis. This reduction in PPT1 weakens its interaction with P300, thereby increasing palmitoylation at C1176 of P300 and protecting P300 from lysosomal degradation. Moreover, palmitoylation inhibits P300 phase separation, maintains its acetyltransferase activity, and ultimately promotes adipogenic differentiation.

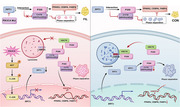

## 1 Introduction

Phosphatidylinositol 3‐kinase catalytic subunit alpha (*PIK3CA*) encodes the p110α catalytic subunit of phosphoinositide 3‐kinase (PI3K). *PIK3CA* oncogenic mutations disrupt the inhibitory interaction between p110α and its regulatory subunit p85α, resulting in constitutive activation of the PI3K–AKT cascade that drives proliferation, survival, and lineage‐specific differentiation [1]. Beyond malignancy, post‐zygotic *PIK3CA* mutations also underlie a spectrum of benign overgrowth disorders collectively designated as *PIK3CA*‐related overgrowth spectrum (PROS) [2]. Facial infiltrating lipomatosis (FIL), a PROS subtype characterized by progressive and maldistributed adipose expansion, provides an instructive human model for dissecting how mutant *PIK3CA* perturbs adipogenic homeostasis [3]. Histologically, mature adipocytes demonstrate excessive hyperplasia and infiltrate adjacent soft tissues, blurring anatomical planes [4]. In 2014, scholars first identified somatic *PIK3CA* mutations in FIL lesions and subsequently demonstrated the same variants in multiple mesenchymal lineages, including bone, muscle, and dermis, confirming its mosaic nature [5, 6]. We subsequently delineated the genomic landscape of the largest Chinese FIL cohort to date, detecting pathogenic *PIK3CA* variants in >80% of patients [7]. Nevertheless, the molecular relays that link mutant *PIK3CA* to dysregulated adipogenesis remain incompletely mapped. Elucidating these downstream effectors will illuminate novel therapeutic vulnerabilities and facilitate the development of mutation‐guided, tissue‐sparing interventions [8].

Aberrant adipogenic commitment of adipose‐derived stem and progenitor cells (ASPCs) constitutes a pivotal driver of FIL initiation and progression, and is intimately linked to the dysregulation of multiple post‐translational modifications (PTMs), including phosphorylation, methylation, ubiquitination, glycosylation and acylation [9, 10]. Palmitoylation is a reversible PTM that covalently attaches a 16‐carbon palmitate to cysteine residues via a thioester linkage, thereby markedly increasing protein hydrophobicity and directing proteins to specific membranes or protein interactomes, consequently dictating subcellular localization and signal transduction [11]. Depalmitoylation, catalyzed chiefly by palmitoyl‐protein thioesterases such as palmitoyl‐protein thioesterase 1 (PPT1), antagonizes this lipidation cycle and profoundly influences cellular behavior [12]. Emerging evidence indicates that PPT1 governs lineage specification in diverse cell types. For instance, PPT1‐null conventional type 1 dendritic cells exhibit an enhanced capacity to prime naïve CD8^+^ T cells into tissue‐resident KLRG1^+^ effector and memory subsets, thereby accelerating tumor clearance [13]. In oocytes, elevated PPT1 activity reduces global S‐palmitoylation, perturbing meiotic maturation and spindle assembly [14]. Moreover, in C2C12 myoblasts, PPT1 sustains lysosomal proteolysis and autophagic flux, prerequisites for efficient myogenic differentiation [15]. Collectively, these observations posit PPT1 as a critical modulator of intracellular signaling and organellar homeostasis during cellular differentiation; nevertheless, its role in adipogenic commitment of ASPCs remains undefined.

Research on liquid‐liquid phase separation (LLPS) has rapidly become a central theme in cell biology. When proteins or non‐coding RNAs reach sufficient concentration they demix from the bulk solution, nucleating micrometer‐scale droplets that behave like liquids [16]. It occurs when biomolecules interact multivalently, forming dense condensates without membranes [17]. Proteins involved usually have intrinsically disordered regions (IDRs) that provide weak, nonspecific interactions, promoting LLPS [18]. This mechanism modulates the spatiotemporal controls of many biological behaviors, such as gene regulation and signal transduction. For example, phase separation of RUNX2 bridges enhancers to the XCR1 promoter, establishing long‐range chromatin contacts that upregulate XCR1 and thereby accelerate osteoblastic differentiation [19]. lncRNA‐MEG3 binds to SUZ12, stabilizes PRC2, facilitates SUZ12 LLPS, and regulates the epigenetic modulation of Fhl3 and ring Rnf128 [20]. Recent studies have shown that ZDHHC7‐mediated palmitoylation in resting cells lowers the threshold for NLRP3 phase separation, priming it to respond to diverse stimuli. However, whether palmitoylation exerts a similar effect on the LLPS of other proteins remains unclear [21].

Here, we demonstrate for the first time that *PIK3CA* activating mutations suppress PPT1 transcription through the PI3K‐AKT‐c‐JUN pathway. The consequent reduction in PPT1 protein attenuates its interaction with P300, thereby increasing Cys1176 palmitoylation of P300. This modification prevents lysosomal degradation of P300 and simultaneously antagonizes its phase separation, thus preserving histone acetyltransferase activity. Sustained P300 activity drives continuous expression of adipogenic genes, ultimately promoting aberrant adipogenesis in FIL.

## 2 Results

### 2.1 Downregulated PPT1 Expression in FIL

**FIGURE 1:**
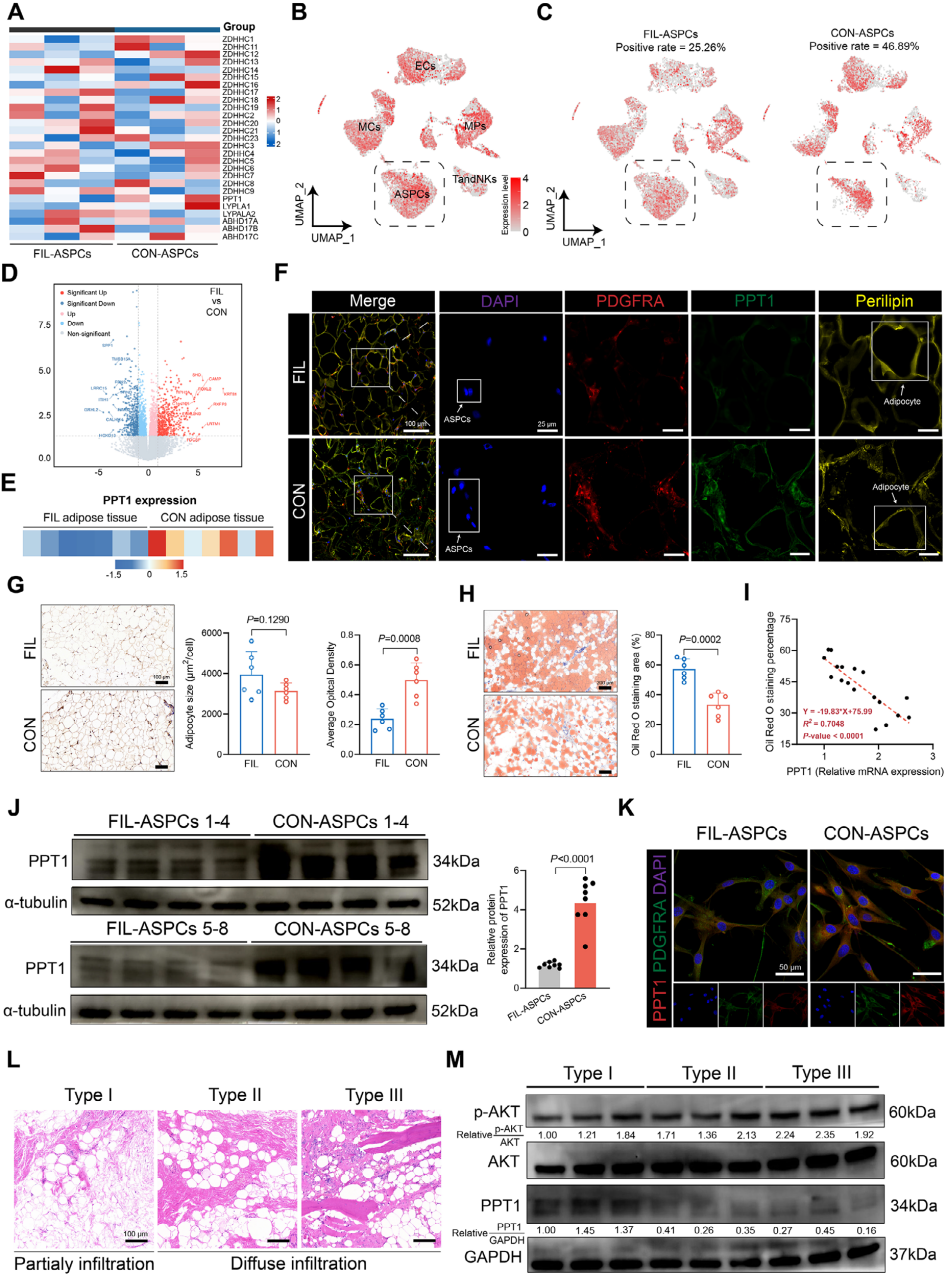
The downregulation of PPT1 in FIL is associated with its severity. (A) The heatmap displays the expression of various palmitoylation‐related genes in FIL‐ASPCs and CON‐ASPCs. (B) Feature plot depicting the expression distribution of PPT1 in cells derived from FIL and CON adipose tissues after the integration of all samples. The expression levels for each cell were colour‐coded and overlaid onto the uniform manifold approximation and projection (UMAP) plot; the dashed box indicated adipose stem and progenitor cells (ASPCs). Macrophages (MPs), mural cells (MCs), T and natural killing cells (TandNKs), endothelial cells (ECs), Single‐cell data were acquired from GSE267777. (C) Feature plots of the distribution of PPT1 expression in ASPCs, split by tissue. Color intensity represents the expression levels of PPT1 in FIL‐ASPCs and CON‐ASPCs. The positive rates of PPT1 in FIL‐ASPCs and CON‐ASPCs were 25.26% and 46.89%, respectively. (D) Volcano plot of significantly upregulated (red) and downregulated (blue) genes identified by comparing FIL and CON adipose tissue. (E) The heatmap shows the expression of PPT1 in FIL and CON adipose tissues. (F) Representative immunofluorescence staining of FIL and CON adipose tissue. (G) Analysis of PPT1 expression in FIL adipose tissues in comparison with CON adipose tissues by immunohistochemistry assay. Scale bar: 100 µm. (H) Oil red O staining and quantitative analysis of the proportion of the oil red O staining area within the whole visual field of adipose tissue from the FIL and CON (representing the area of the lipid droplets). Scale bar: 200 µm. (I) Correlation analysis of the relative mRNA expression of PPT1 and the Oil Red O staining area. Significance was determined by Pearson correlation analysis (*R^2^* =0.4843, *p* = 0.0003). (J) Western blot showing PPT1 expression relative to GAPDH expression in FIL‐ASPCs and CON‐ASPCs. (K) Immunofluorescence analysis of PPT1 protein level. (L) Hematoxylin‐Eosin (H&E) staining of adipose tissue isolated from FIL patients with different classification subtypes. (M) Western blot of PPT1 in adipose tissue isolated from FIL patients with different classification subtypes. Data were analyzed by unpaired two‐sided Student's *t* tests (G, H, and J) and were presented as mean ± SD.

To delineate which palmitoylation‐related enzymes might be implicated in FIL, we first analyzed our single‐cell RNA‐seq atlas generated from FIL patients and control (CON) individuals (GEO accession GSE267777). We specifically interrogated the expression profile of all palmitoylation‐associated genes within the ASPCs cluster. Notably, ZDHHC12 and PPT1 were among the most significantly downregulated transcripts in FIL‐ASPCs compared with CON‐ASPCs (Figure 1A). To validate these in silico findings, we isolated ASPCs from additional FIL and CON samples and confirmed their purity by flow cytometry (Figure S1A,B). RT‐qPCR revealed that only PPT1 mRNA levels were reproducibly reduced in FIL‐ASPCs, whereas ZDHHC12 expression did not differ significantly (Figure S2A). Consequently, subsequent studies focused on PPT1.

The feature plot demonstrating the expression distribution of PPT1 in FIL and CON adipose tissue was superimposed onto the unsupervised uniform manifold approximation and projection (UMAP) clustering plot, revealing that PPT1 was ubiquitously expressed across multiple cell types (Figure 1B). Notably, its abundance was markedly elevated in CON‐ASPCs, as evidenced by a higher frequency of PPT1‐positive ASPCs (Figure 1C). To interrogate the transcriptional landscape, we performed high‐depth RNA‐seq on adipose tissue biopsies from seven FIL subjects and seven age‐matched controls (GEO: GSE300520). A total of 540 genes were upregulated and 436 downregulated in FIL adipose tissue (|log2FC| ≥ 0.58, FDR < 0.05; Figure 1D). Gene ontology (GO) enrichment analysis revealed that the upregulated gene set was significantly enriched for pathways governing cell differentiation (Figure S2B). Strikingly, PPT1 transcript levels were substantially reduced in FIL adipose tissue (Figure 1E). To delineate PPT1 expression under native conditions, we conducted multicolor immunofluorescence staining. Both ASPCs and mature adipocytes in FIL displayed a pronounced reduction in PPT1 protein content relative to controls (Figure 1F). Immunohistochemical corroboration further demonstrated diminished PPT1 signal intensity throughout FIL specimens, concomitant with adipocyte hypertrophy (Figure 1G). Oil Red O staining revealed exaggerated lipid accumulation within FIL adipose tissue (Figure 1H), and correlation analysis disclosed a significant inverse correlation between PPT1 mRNA levels and lipid area (*R* ^2^ = 0.7048, *p* < 0.01; Figure 1I).

Subsequently, we quantified PPT1 protein abundance in primary ASPCs. Western blot and immunofluorescence revealed a pronounced reduction in PPT1 levels in FIL‐ASPCs relative to CON‐ASPCs (Figure 1J,K, S2C). We have previously established a histology‐based stratification of FIL severity: type I was characterized by focal, minimal adipose infiltration, whereas types II–III exhibited progressive, diffuse adipose replacement (Figure 1L) [22]. Western blot demonstrated an inverse relationship between pathological severity and PPT1 protein content, with the lower expression observed in type‐III and type‐II specimens compared with type‐I (Figure 1M). Collectively, these data demonstrated that PPT1 was consistently downregulated in both FIL adipose tissue and resident ASPCs, and its abundance correlated inversely with disease severity.

### 2.2 PIK3CA Mutation Suppressed PPT1 Expression Through Activation of c‐JUN

**FIGURE 2:**
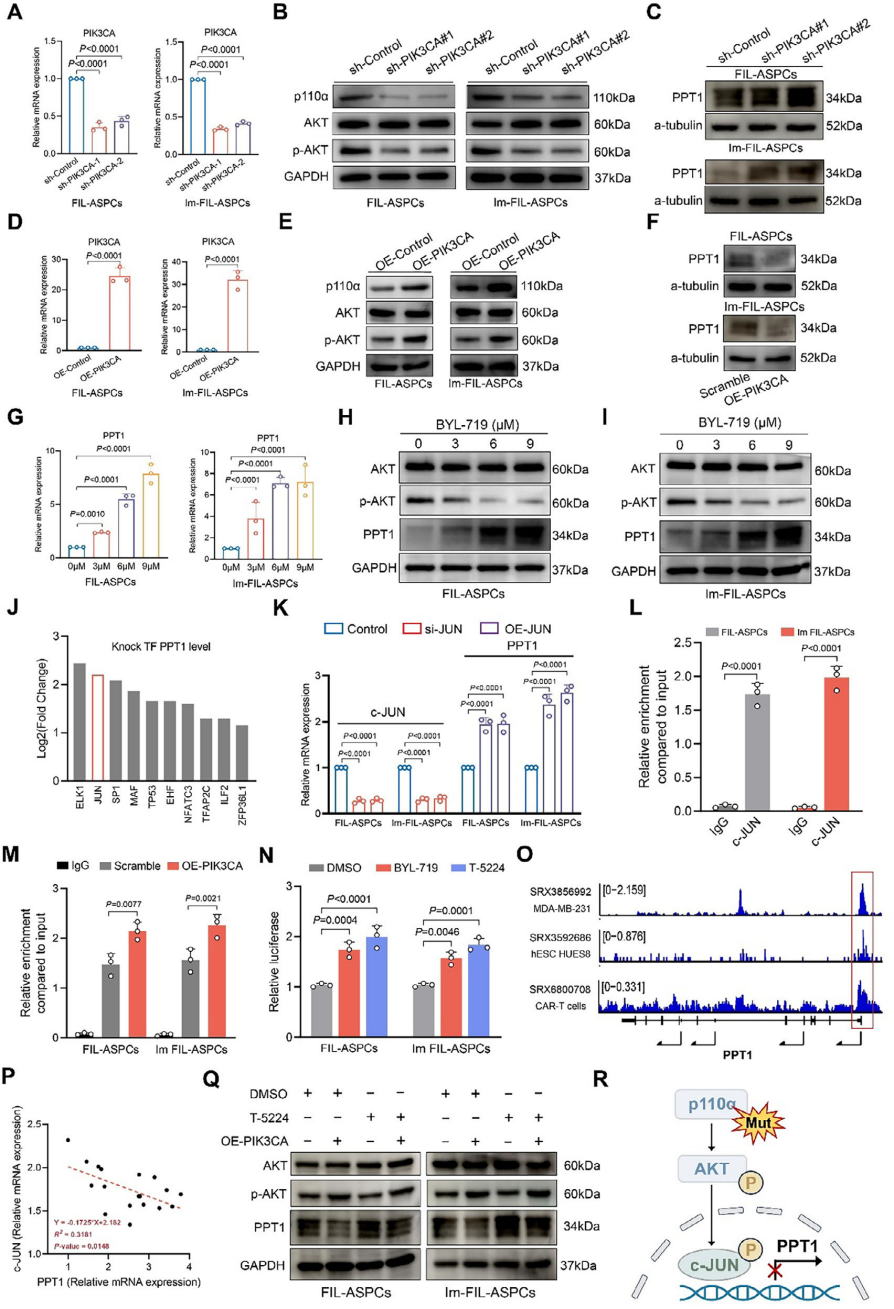
PIK3CA mutation inhibited PPT1 expression via c‐JUN. (A) RT‐qPCR results showed the silencing of PIK3CA after lentivirus transfection. GAPDH mRNA served as a reference standard. (B) Western blot analysis of proteins downstream of the PI3K‐AKT pathway in PIK3CA‐knockdown FIL‐ASPCs and Im FIL‐ASPCs. GAPDH protein levels were used as a reference standard. (C) Western blot analysis of PPT1 protein level in PIK3CA‐knockdown FIL‐ASPCs and Im FIL‐ASPCs. (D) RT‐qPCR results showed the overexpression of PIK3CA after lentivirus transfection. (E) Western blot analysis of proteins downstream of the PI3K‐AKT pathway in PIK3CA‐overexpression FIL‐ASPCs and Im FIL‐ASPCs. (F) Western blot analysis of PPT1 protein level in PIK3CA‐overexpression FIL‐ASPCs and Im FIL‐ASPCs. (G) RT‐qPCR results showed PPT1 mRNA level in FIL‐ASPCs and Im FIL‐ASPCs treated with BYL‐719. (H, I) Western blot showed PPT1 and p‐AKT level in FIL‐ASPCs and Im FIL‐ASPCs treated with BYL‐719. (J) The analysis of the transcription factors that target PPT1 based on the KnockTFv2 platform. (K) RT‐qPCR assay for the mRNA expression level of c‐JUN and PPT1 in FIL‐ASPCs and Im FIL‐ASPCs transfected with c‐JUN siRNA. (L) ChIP‐qPCR analysis of PPT1 by using the IgG or c‐JUN antibodies in PIK3CA‐overexpression FIL‐ASPCs and Im FIL‐ASPCs. (M) ChIP‐qPCR analysis of PPT1 by using the IgG or c‐JUN antibodies in PIK3CA‐overexpression FIL‐ASPCs and Im FIL‐ASPCs. (N) The dual‐luciferase reporter assay for the promoter region binding affinity of PPT1 in FIL‐ASPCs treated with BYL‐719 or T‐5224. (O) c‐JUN sequencing data from ChIP‐Atlas dataset indicated that there were binding peaks in the promoter region of PPT1. (P) Correlation analysis of the relative mRNA expression of PPT1 and the Oil Red O staining area. Significance was determined by Pearson correlation analysis (*R^2^* = 0.3181, *p* = 0.0148). (Q) Western blot analysis of FIL‐ASPCs and Im FIL‐ASPCs treated with DMSO/T‐5224 and infected with PI3KCA‐overexpression lentivirus. (R) Model delineating the mechanism that PIK3CA mutations suppressed PPT1 expression through PI3K‐AKT‐c‐JUN pathway. Experiments were independently replicated at least three times with similar results (biological replicates). Data were analyzed by unpaired two‐sided Student's *t* tests (D and L) or one‐way ANOVA (A, G, K, M, and N) and were presented as mean ± SD with three replicate experiments.

Somatic PIK3CA mutation constitutively activate the PI3K‐AKT axis, heightening AKT phosphorylation and thereby reprogramming the transcriptional landscape and cellular phenotype [23]. To determine whether mutant PIK3CA represses PPT1 expression, we referred to methods from previous literature and first silenced PIK3CA in both primary FIL‐ASPCs and the previously established immortalized FIL‐ASPC cell line (Im FIL‐ASPCs) [24, 25, 26]. The primary FIL‐ASPCs retain the patient‐specific genetic background and pathophysiological context, providing clinically relevant insights. In parallel, the Im FIL‐ASPCs, which stably maintain the key pathogenic features of FIL including the PIK3CA mutation and enhanced adipogenic potential, ensure experimental reproducibility and facilitate genetic manipulations [24]. This intervention concordantly diminished AKT phosphorylation (Figure 2A,B). RT‐qPCR and immunoblot analyses revealed that PIK3CA knock‐down markedly upregulated PPT1 mRNA and protein levels (Figure S3A; Figure 2C). Conversely, lentivirus‐mediated PIK3CA overexpression attenuated PPT1 expression (Figure 2D–F; Figure S3B). Pharmacological blockade of PI3K with BYL719 dose‐dependently elevated PPT1 mRNA and protein abundance (Figure 2G–I). The hotspot oncogenic variants PIK3CA^H1047R^ and PIK3CA^E542K^ exhibited stronger oncogenic capacity. Immunohistochemistry of FIL adipose tissue demonstrated that specimens harboring these hotspot mutations displayed lower PPT1 level than those carrying non‐hotspot PIK3CA alterations (Figure S3C).

To elucidate the mechanism by which PIK3CA mutations downregulate PPT1, we interrogated the KnockTF platform for transcription factors predicted to repress PPT1. Among the top 10 candidate repressors, c‐JUN is a well‐established downstream target of the PI3K‐AKT pathway (Figure 2J) [27]. Genetic depletion of c‐JUN robustly increased PPT1 mRNA and protein abundance, whereas its enforced expression markedly attenuated PPT1 levels in FIL‐ASPCs (Figure 2K; Figure S3D–F). Chromatin immunoprecipitation (ChIP)‐qPCR verified direct occupancy of c‐JUN at the PPT1 promoter (Figure 2L), and this occupancy was significantly enhanced upon PIK3CA overexpression (Figure 2M). Dual‐luciferase reporter assays revealed that either PI3K inhibition or c‐JUN blockade markedly elevated PPT1 promoter activity (Figure 2N). Analysis of ChIP‐Atlas meta‐database further confirmed c‐JUN binding to the PPT1 promoter across multiple cell lineages (Figure 2O). Consistently, correlation analyses disclosed a significant inverse correlation between c‐JUN and PPT1 mRNA levels (Figure 2P). Importantly, pharmacologic inhibition of c‐JUN partially rescued PIK3CA‐driven suppression of PPT1 (Figure 2Q). Collectively, these data establish that oncogenic PIK3CA signaling transcriptionally repressed PPT1 via c‐JUN‐mediated promoter inactivation (Figure 2R).

### 2.3 PPT1 Inhibited Adipogenesis in a Palmitoylation‐Dependent Manner

**FIGURE 3:**
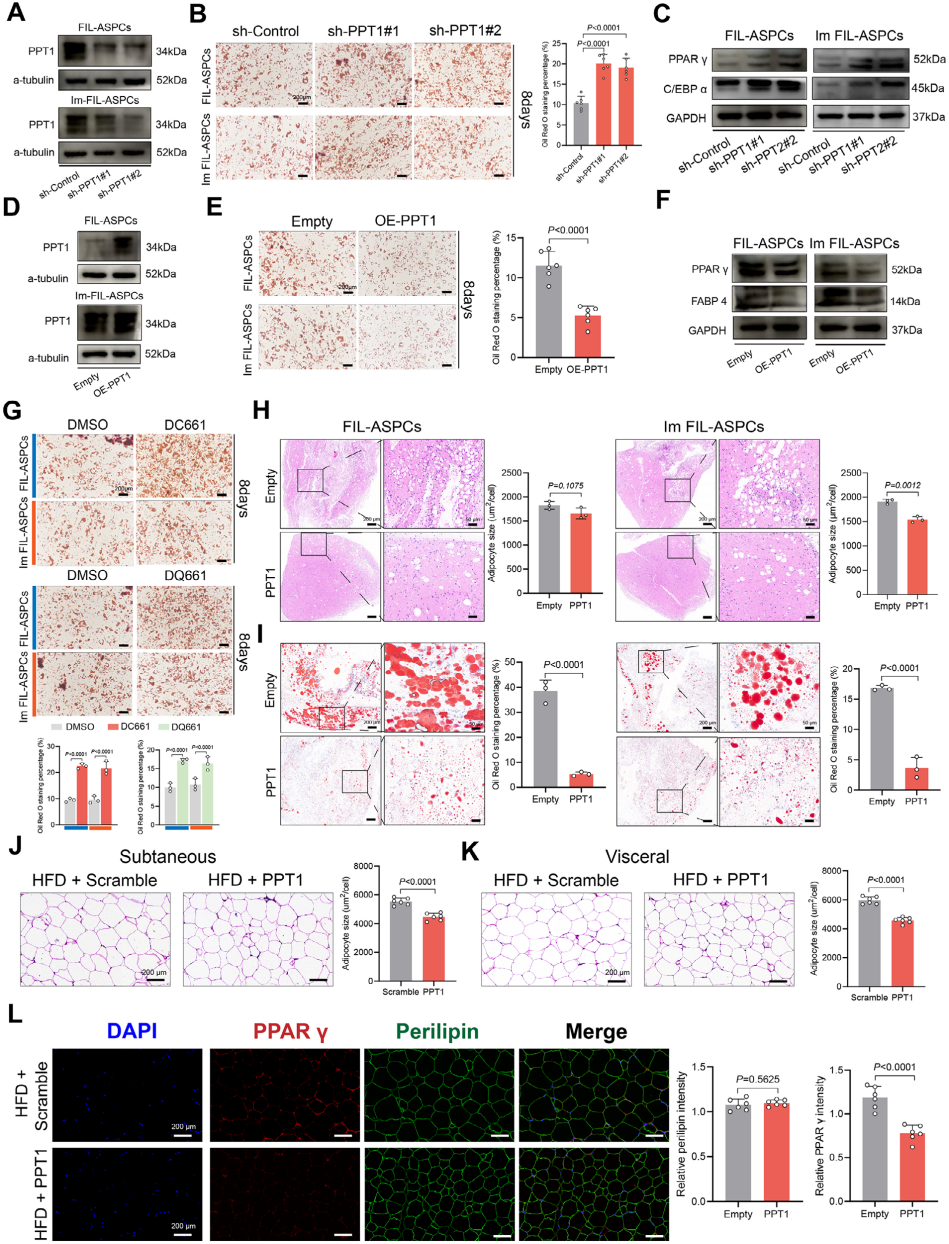
PPT1 inhibited adipogenesis both in vitro and in vivo. (A) Western blot showing PPT1 expression relative to GAPDH expression in FIL‐ASPCs and Im FIL‐ASPCs upon FTO knockdown. (B) Oil Red O staining was used to evaluate the effect of PPT1 knockdown on lipid droplet accumulation at day 8 of adipogenesis. Scale bar: 200 µm. (C) Western blot analysis showed that the protein levels of PPAR γ and C/EBP α in FIL‐ASPCs and Im FIL‐ASPCs upon PPT1 knockdown compared with the wild type at day 3 of adipogenesis. (D) Western blot showing PPT1 expression relative to GAPDH expression in FIL‐ASPCs and Im FIL‐ASPCs upon PPT1 overexpression. (E) Oil Red O staining was used to evaluate the effect of PPT1 overexpression on lipid droplet accumulation at day 8 of adipogenesis. Scale bar: 200 µm. (F) Western blot analysis showed that the protein levels of PPAR γ and FABP 4 in FIL‐ASPCs and Im FIL‐ASPCs upon PPT1 expression compared with the control group at day 3 of adipogenesis. (G) Oil Red O staining was used to evaluate the effect of DC661 and DQ61 on lipid droplet accumulation at day 8 of adipogenesis. Scale bar: 200 µm. (H) H&E staining of Matrigel implants collected on day 28 and quantification of adipocytes size. Scale bar: left: 200 µm, right: 50 µm. (I) Oil Red O staining of Matrigel implants from three groups and quantification of Oil red O staining area. Scale bar: left: 200 µm, right: 50 µm. (J, K) H&E staining showed the size of subcutaneous (J) and visceral (K) adipocytes from different groups. Scale bar: 200 µm. (L) Perilipin and PPAR γ were visualized by immunofluorescence staining. Scale bar: 200 µm. Data were analyzed by unpaired two‐sided Student's *t* tests (E, H, I, J, K, and L) or one‐way ANOVA (B) and were presented as mean ± SD.

To define the functional contribution of PPT1 to adipogenic commitment of FIL‐ASPCs, we first generated stable PPT1 knockdown lines using lentiviral short‐hairpin RNAs (Figure 3A). Upon 8 days of adipogenic induction, both primary and Im FIL‐ASPCs exhibited markedly accelerated lipid accumulation, as gauged by Oil Red O staining (Figure 3B). Consistently, PPT1 knockdown significantly upregulated the master adipogenic transcription factor PPARγ and C/EBP α at protein levels (Figure 3C). Conversely, lentivirus‐mediated overexpression of PPT1 suppressed lipid droplet formation and PPARγ/FABP4 induction (Figure 3D–F). Moreover, selective PPT1 inhibitors DC661 and DQ661 dose‐dependently potentiated adipocyte differentiation (Figure 3G; Figure S4A,B).

Given that PPT1 exerted its principal enzymatic function via depalmitoylation, we postulated that its anti‐adipogenic activity might rely on this catalytic property. Indeed, co‐treatment with the global palmitoylation blocker 2‐bromopalmitate (2‐BP) completely reversed the pro‐adipogenic phenotype elicited by PPT1 silencing (Figure S4C,D). Previous study revealed that two residues of PPT1, Ser115 and His289, are essential for its catalytic activity [28]. Cross‐species alignment confirmed relative conservation of these catalytic amino acids (Figure S4E). Therefore, we constructed two PPT1 mutants (S115A and H289A) without significant catalytic activity and then ectopically transfected FIL‐ASPCs and Im FIL‐ASPCs with plasmids expressing wild‐type PPT1 and the two mutants. Expression of the two mutants considerably disrupted the inhibitory effect in adipogenesis of FIL‐ASPCs (Figure S4F–H).

To investigate the in vivo effect of PPT1 in adipogenesis, we first established a xenograft model by subcutaneously implanting FIL‐ASPCs and Im FIL‐ASPCs with or without PPT1 overexpression. Grafts were harvested 4 weeks post‐implantation [26]. Hematoxylin‐eosin (H&E) staining revealed a pronounced reduction in mature adipocyte content within PPT1‐overexpressing implants (Figure 3H), and Oil red O staining confirmed an almost complete absence of large lipid droplets (Figure 3I). To directly assess the role of PPT1 in physiological adipogenesis, we constructed an adeno‐associated virus serotype‐8 (AAV8) vector in which the PPT1 coding sequence was placed under transcriptional control of the adipocyte‐specific FABP4 promoter. Mice maintained on a high‐fat diet (HFD) received a single systemic tail‐vein injection of either AAV8‐FABP4‐PPT1‐eGFP (HFD‐PPT1) or AAV8‐FABP4‐Scramble‐eGFP (HFD‐Scramble) and were sacrificed 6 weeks later for phenotypic analysis (Figure S5A). Immunofluorescence microscopy documented robust co‐localization of eGFP with perilipin‐positive adipocytes, validating efficient adipocyte‐restricted transduction (Figure S5B). Immunoblotting of isolated adipocytes confirmed a marked elevation of PPT1 protein in HFD‐PPT1 animals relative to controls (Figure S5C). Phenotypically, HFD‐PPT1 mice exhibited a visibly leaner habitus (Figure S5D). Morphometric analysis of H&E‐stained sections demonstrated a significant reduction in both subcutaneous and visceral adipocyte diameter in HFD‐PPT1 mice compared with HFD‐Scramble group (Figure 3J,K). Consistent with these observations, immunofluorescence quantification revealed a decrease in PPAR γ signal intensity within the adipose tissue of HFD‐PPT1 animals (Figure 3L). Collectively, these in vivo data corroborated our in vitro findings and indicated that PPT1 suppressed adipocyte differentiation, at least in part, through its depalmitoylating activity.

### 2.4 P300 was Identified as a Novel PPT1‐Interacting Protein

**FIGURE 4:**
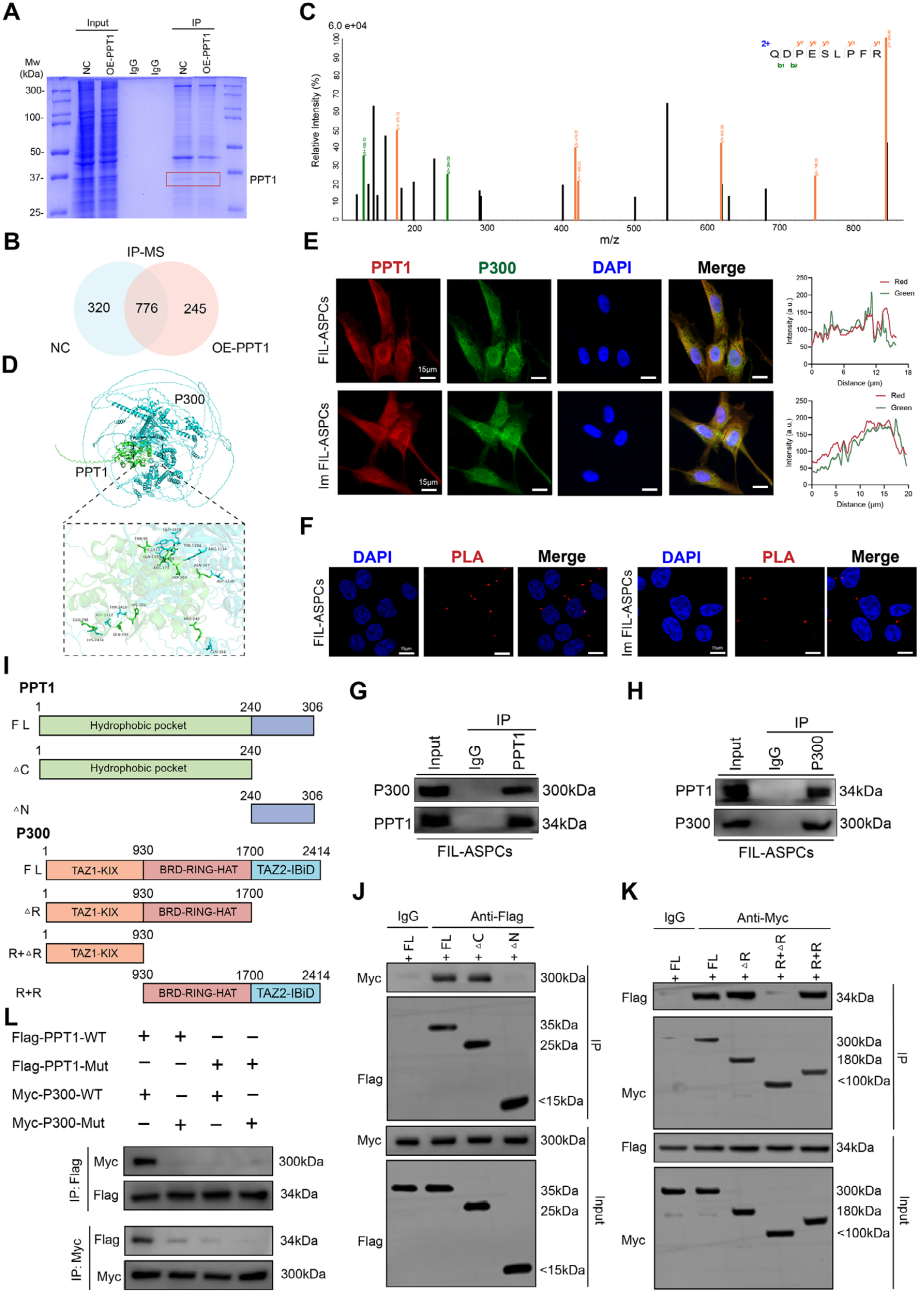
PPT1 physically interacted with P300. (A) Gel electrophoresis was performed after using PPT1 antibody for PPT1‐immunoprecipitation, then Coomassie brilliant blue staining was conducted. The red frame showed the PPT1 and P300 predicted based on molecular weight. (B) The results of immunoprecipitation‐mass spectrometry (IP‐MS) analysis in Im FIL‐ASPCs with or without PPT1 overexpression. (C) Mass spectrometry analysis of a peptide derived from PPT1‐immunoprecipitates to show the potential interaction between PPT1 and P300. (D) The predicted model of the protein complex was obtained through computation via the AlphaFold3 server. The output file was further analyzed with PyMOL, and detailed images of the binding interface between the two proteins were generated. (E) PPT1 and P300 were detected by immunofluorescence staining in FIL‐ASPCs and Im FIL‐ASPCs (left). The co‐localization analysis was performed by Image J (right). The red line indicated the region used for measuring the colocalization signal. (F) Proximity ligation assays (PLA) were performed using anti‐PPT1 and anti‐P300 antibodies in FIL‐ASPCs and Im FIL‐ASPCs. Red signals represent PLA signals, indicating the proximity of the targeted proteins, while blue signals denote the cell nuclei. (G, H) FIL‐ASPCs cellular lysates were analyzed by Co‐IP followed by western blotting. (I) Schematic representation of PPT1 and P300 truncations. (J) IP and western blot assay of the interaction between FLAG‐tagged truncated PPT1 and Myc‐tagged P300 in HEK293T cells. Cell extracts were IP with an anti‐Flag antibody. (K) IP and western blot assay of the interaction between FLAG‐tagged PPT1 and Myc‐tagged truncated P300 in HEK293T cells. Cell extracts were IP with an anti‐Myc antibody. (L) IP and western blot assay show the interactions between FLAG‐tagged mutated PPT1 and Myc‐tagged mutated P300 in HEK293T cells. The point mutations were designed according to the scheme showed in Figure S7E.

PPT1 typically exerts its regulatory effects via depalmitoylation of downstream substrates rather than by directly remodeling cellular phenotypes. Given that PPT1 lacks canonical transcription‐factor activity, we next sought to identify the proximal substrates through which it modulated adipogenesis. Coomassie‐brilliant‐blue staining of PPT1 immunoprecipitates separated by SDS‐PAGE revealed distinct, molecular‐weight‐specific bands that were enriched in FIL‐ASPCs overexpressing PPT1 (Figure 4A). FIL‐ASPCs with or without PPT1‐overexpression were selected to make immunoprecipitation‐mass spectrometry (IP‐MS) analysis, and 776 proteins were identified as potential PPT1‐interacting proteins (Figure 4B). IP‐MS analysis indicated PPT1 might interact with P300 (Figure 4C). Prior studies have implicated P300 as an obesity‐associated marker that potentiates CEBPA expression, positioning it as a putative node in FIL‐ASPC adipogenic regulation [29, 30].

We first asked whether PPT1 physically associates with P300. In silico docking predicted a compatible interface involving multiple amino‐acid contacts distributed across both proteins (Figure 4D, Figure S7A). To evaluate the thermodynamic and kinetic stability of the PPT1‐P300 complex, we conducted 10‐ns all‐atom molecular‐dynamics (MD) simulations in explicit solvent. Root‐mean‐square deviation (RMSD) versus time indicated rapid structural relaxation within the first 1.5 ns (RMSD = 2.0 nm), followed by equilibration within a narrow 2.2–2.8 nm envelope for the remainder of the trajectory (Figure S6A). Root‐mean‐square fluctuation (RMSF) analysis revealed that P300 harbors both a stable core and several flexible loops‐an architecture consistent with its multifunctional transcriptional regulatory role‐whereas the PPT1 thioesterase domain remained globally rigid throughout the simulation (Figure S6B,C). The radius of gyration (Rg) decreased monotonically before plateauing, signifying condensation into a compact, well‐folded heterodimer (Figure S6D). Concomitantly, solvent‐accessible surface area (SASA) declined progressively, reflecting expulsion of water molecules and closer packing at the inter‐protein interface (Figure S6E). Intermolecular hydrogen bonds fluctuated between 10 and 20, but never collapsed, indicating a persistently engaged contact surface (Figure S6F). The minimum inter‐residue distance hovered around 0.17 nm (1.7 Å) for >80% of the trajectory, corroborating intimate, stable association (Figure S6G). Finally, free‐energy landscape mapping converged on a single low‐energy basin, underscoring the robustness of the PPT1‐P300 complex (Figure S6H,I). Collectively, the MD data affirmed that PPT1 and P300 formed a structurally cohesive and energetically favorable complex, providing a plausible molecular framework for subsequent functional interrogation.

Immunofluorescence staining revealed co‐localization of PPT1 and P300 (Figure 4E). The proximity ligation assay (PLA) indicated the transient interaction of endogenous PPT1 with P300 (Figure 4F). Co‐IP confirmed flag‐tagged PPT1 and myc‐tagged P300 were exogenously overexpressed in 293T cells, and their interaction was also observed (Figure S7B,C). Consistently, endogenous PPT1 also interacted with P300 in FIL‐ASPCs and Im FIL‐ASPCs (Figure 4G,H, Figure S7D). Based on their structures, the full‐length PPT1 protein was divided into two fragments, and the P300 protein was cut into three segments (Figure 4I). Co‐IP mapping revealed that the hydrophobic pocket of PPT1 (residues 1‐240) is necessary for P300 interaction, with the cognate binding region of P300 localized between residues 930 and 1700 (Figure 4J–K). Reverse Co‐IP assays corroborated these findings, demonstrating reciprocal capture of PPT1 (1‐240) by P300 (930‐1700) (Figure S7F,G). Docking simulations further indicated that residues 174 and 198 of PPT1 may interface with residues 1195 and 1240 of P300 (Figure S7E). These mutations, whether introduced individually to the PPT1‐binding site or the P300‐binding site, or simultaneously to both, effectively disrupted the interaction between PPT1 and P300 (Figure 4L). These data confirmed P300 as a novel PPT1 substrate.

### 2.5 PPT1‐Mediated Depalmitoylation Promoted P300 Degradation via the Lysosomal Pathway

**FIGURE 5:**
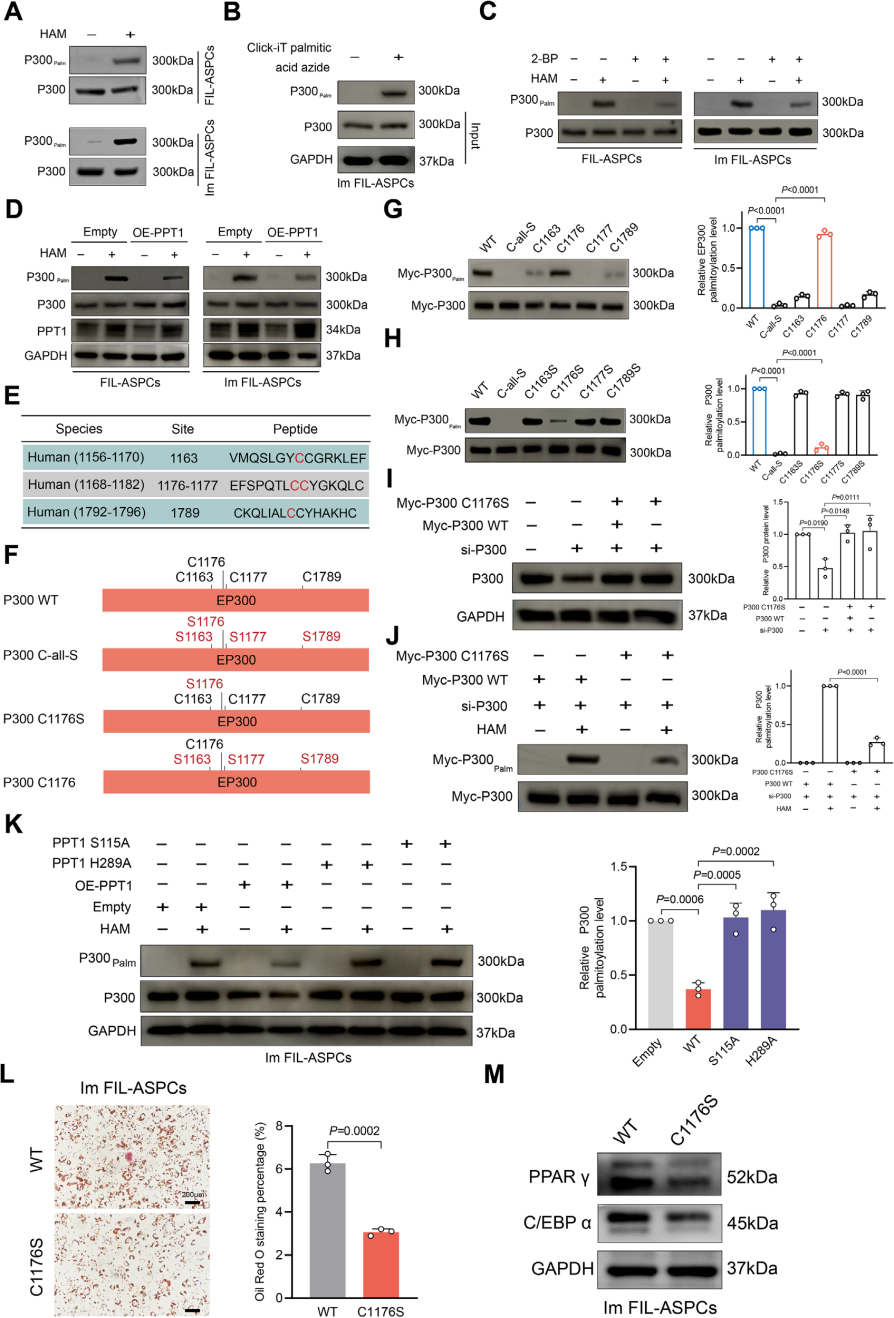
PPT1 modulated the palmitoylation of P300 in FIL‐ASPCs. (A) FIL‐ASPCs and Im FIL‐ASPCs were subjected to an ABE assay and immunoblot analysis. (B) FIL‐ASPCs with or without ALK‐C16 treatment were harvested for Click‐IT reaction and streptavidin pulldown. (C) P300 palmitoylation levels in the FIL‐ASPCs and Im FIL‐ASPCs were analyzed using ABE and immunoblot assays after 2‐BP treatment. (D) P300 palmitoylation levels in the FIL‐ASPCs and Im FIL‐ASPCs with or without PPT1 overexpression were analyzed using ABE and immunoblot assays. (E) The amino acid sequences flanking the top four P300 palmitoylation sites predicted by CSS‐Palm 4.0. (F) Schematic illustration of P300 mutants. (G, H) Immunoblotting analysis of P300 palmitoylation by ABE assay in HEK293T cells transfected with plasmids encoding WT P300 or its indicated mutants. (I) Western blot assay of HEK293T cells infected with P300 WT/C1176S after endogenous P300 knockdown. (J) P300 palmitoylation was detected by ABE assay of HEK293T cells infected with P300 WT/C1176S after endogenous P300 knockdown. (K) ABE and Western blot analysis of Im FIL‐ASPCs infected with PPT1 WT/S115A/H289A plasmids. (L) Oil Red O staining showing lipid accumulation in Im FIL‐ASPCs transfected with P300 WT/C1176S after 8 days of adipogenic induction. (M) Western blot analysis of PPAR γ and C/EBP α expression in Im FIL‐ASPCs transfected with P300 WT/C1176S after 3 days of adipogenic induction. Experiments were independently replicated at least three times with similar results (biological replicates). Data were analyzed by unpaired two‐sided Student's *t* tests (G, H, and J) or one‐way ANOVA (I and K) and were presented as mean ± SD with three replicate experiments.

We next investigated whether PPT1 modulates P300 activity by altering its palmitoylation status. Acyl‐biotin exchange (ABE) and click‐chemistry assays demonstrated that P300 is indeed S‐palmitoylated in FIL‐ASPCs (Figure 5A,B). Either pharmacological blockade of global palmitoylation with 2‐BP or enforced expression of wild‐type PPT1 markedly reduced P300 palmitoylation (Figure 5C,D). To identify the palmitoylation sites, we performed in silico prediction with CSS‐Palm 4.0 and ranked the top four scoring residues (Figure 5E). Sequential mutagenesis revealed that simultaneous substitution of all four cysteines to serines (C‐all‐S) completely abolished P300 palmitoylation (Figure 5F,G). Systematic reversion of each serine back to cysteine pinpointed C1176 as the principal palmitoylation site, since only the C1176 single‐cysteine revertant restored detectable palmitoylation to levels comparable to wild‐type P300 (Figure 5G). Consistently, the C1176S point‐mutant exhibited a profound loss of palmitoylation (Figure 5H–J). Importantly, the PPT1‐mediated reduction in P300 palmitoylation was strictly dependent on PPT1 catalytic activity: overexpression of enzymatically dead PPT1 mutants (S115A or H289A) failed to decrease P300 palmitoylation (Figure 5K). Functionally, prevention of P300 palmitoylation‐either by expression of the C1176S mutant significantly impaired adipogenesis of Im‐FIL‐ASPCs, as evidenced by diminished lipid accumulation and attenuated expression of PPARγ and C/EBP α (Figure 5L,M). These data indicated C1176 palmitoylation as a critical PTM that licensed P300 pro‐adipogenic activity.

**FIGURE 6:**
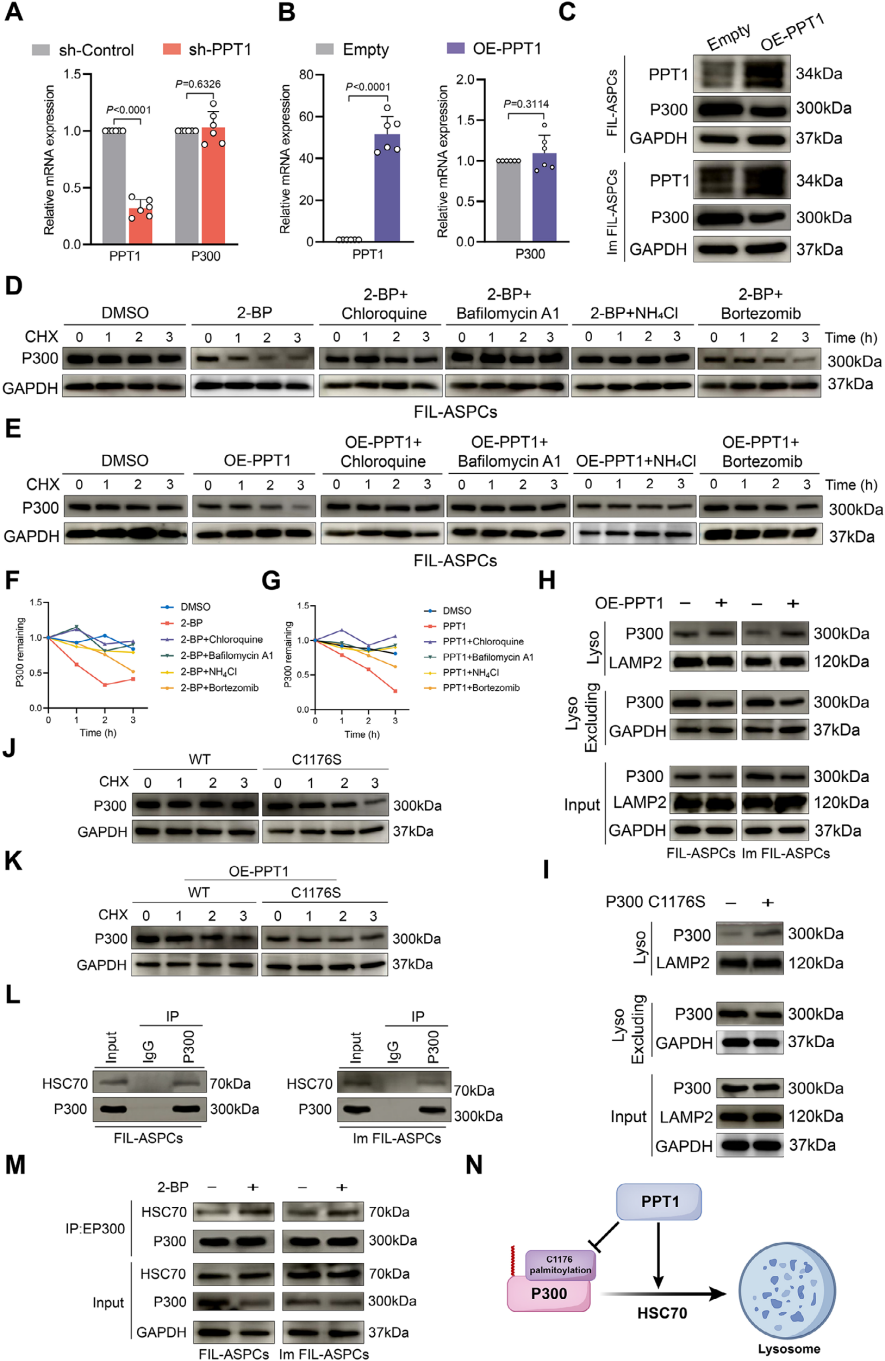
PPT1 promoted the degradation of P300 via depalmitoylation on Cys1176. (A) RT‐qPCR analysis for the mRNA expression level of PPT1 and P300 in FIL‐ASPCs transfected with PPT1 shRNAs. (B) RT‐qPCR analysis for the mRNA expression level of PPT1 and P300 in PPT1‐overexpression FIL‐ASPCs. (C) Western blot analysis for the expression of PPT1 and P300 in FIL‐ASPCs and Im FIL‐ASPCs after PPT1 overexpression. (D) FIL‐ASPCs were subjected to CHX‐chase analysis in the absence or presence of the 2‐BP, combined with lysosome‐blocking agents (chloroquine, bafilomycin A1, or NH_4_Cl) or the proteasome inhibitor bortezomib. (E) FIL‐ASPCs with or without PPT1 overexpression were subjected to CHX‐chase analysis, combined with lysosome‐blocking agents (chloroquine, bafilomycin A1, or NH_4_Cl) or the proteasome inhibitor bortezomib. (F) Quantification of the intensity measured by the relative level of P300 remaining in (D). (G) Quantification of the intensity measured by the relative level of P300 remaining in (E). (H) Cellular fractions were subjected to western blot to compare P300 abundance in lysosomal and extra‐lysosomal compartments between FIL‐ASPCs and Im FIL‐ASPCs, either with or without PPT1 overexpression. (I) Cellular fractions were subjected to Western blot to compare P300 abundance in lysosomal and extra‐lysosomal compartments in HEK293T cells transfected with P300 WT/C1176S. (J) The degradation of P300 WT/C1176S in HEK293T cells transfected with P300 WT/C1176S plasmids was evaluated by CHX‐chase assay. (K) The degradation of P300 WT/C1176S in PPT1‐overexpression HEK293T cells transfected with P300 WT/C1176S plasmids was evaluated by CHX‐chase assay. (L) Co‐IP assays were performed to determine whether P300 interacted with HSC70 in FIL‐ASPCs and Im FIL‐ASPCs. (M) Co‐IP assays were performed to determine whether P300 interacted with HSC70 in FIL‐ASPCs and Im FIL‐ASPCs treated with 2‐BP. (N) Mechanistic model illustrating that PPT1 depalmitoylation P300 at C1176, thereby enhancing its interaction with HSC70 and subsequent chaperone‐mediated delivery to lysosomes for degradation. Data were analyzed by unpaired two‐sided Student's *t* tests (A and B) and were presented as mean ± SD with three replicate experiments.

To comprehensively elucidate the functional consequences of PPT1‐mediated P300 depalmitoylation, we first assessed whether this PTM influenced P300 subcellular distribution. Nuclear/cytoplasmic fractionation assays demonstrated that neither knock‐down nor overexpression of PPT1 altered the relative nuclear versus cytoplasmic abundance of P300 (Figure S8A,D). Likewise, RT‐qPCR revealed that modulation of PPT1 had no impact on P300 mRNA levels (Figure 6A,B; Figure S8B,C). In contrast, ectopic expression of wild‐type PPT1 led to a measurable reduction in total P300 protein (Figure 6C). Previous studies have implicated palmitoylation in the control of protein degradation [31]. We therefore hypothesized that loss of P300 palmitoylation might accelerate its turnover. Indeed, either 2‐BP treatment or PPT1 overexpression accelerated P300 degradation, an effect that was rescued by lysosomal inhibitors (chloroquine, bafilomycin A1, or NH_4_Cl) but not by the proteasomal blocker bortezomib (Figure 6D–G; Figure S8E–H). Immunoblot analysis of subcellular fractions further revealed that PPT1 overexpression or transfection of P300 C1176S mutant increased the abundance of P300 within the lysosomal compartment (Figure 6H–I). Although P300 C1176S mutation accelerated P300 degradation; however, this destabilization was largely abrogated when the mutant was expressed in Im‐FIL‐ASPCs overexpressing PPT1 (Figure 6J–K). These results indicated that palmitoylation at C1176 protected P300 from lysosomal degradation.

Chaperone‐mediated autophagy (CMA) is a major route for the proteolytic degradation of cytosolic proteins by lysosomes [32]. The molecular chaperone HSC70 recognizes substrates that contain KFERQ‐like motifs and delivers them across the lysosomal membrane for luminal degradation. A previous study showed that palmitoylation of HDAC8 decreases its association with HSC70, thereby partially protecting the protein from lysosomal breakdown [33]. Analysis with KFERQ Finder v0.8 revealed multiple KFERQ motifs in P300 (Figure S8I). Co‐immunoprecipitation confirmed an endogenous interaction between P300 and HSC70 (Figure 6L), and treatment with the palmitoylation inhibitor 2‐BP or transfected with P300 C1176S markedly increased this association (Figure 6M, Figure S9A). Moreover, siRNA‐mediated knockdown of HSC70 partially reversed the 2‐BP‐induced reduction in P300 protein levels (Figure S9B). To evaluate whether macroautophagy or microautophagy participates in P300 turnover, we performed Co‐IP assays and observed no interaction between P300 and the macroautophagy adaptor P62 or the microautophagy core protein VPS4A (Figure S9C,D). Consistently, treatment with the macroautophagy inhibitor 3‐methyladenine failed to rescue the P300 downregulation induced by 2‐BP administration (Figure S9E). Collectively, these data indicated that palmitoylation at C1176 of P300 weakened its interaction with HSC70, partially shielded the protein from CMA‐dependent lysosomal degradation, and thereby elevated P300 abundance (Figure 6N).

### 2.6 P300 Promoted Adipogenesis by Orchestrating Chromatin Accessibility and Epigenetic Remodeling

The functional relevance of P300 in FIL remains undefined. We first investigated whether PPT1 regulated adipogenesis through P300. P300 protein was markedly elevated in FIL tissues and inversely correlated with PPT1 abundance (Figure S10A,B), consistent with previous observation that PPT1‐dependent depalmitoylation targeted P300 to lysosomal degradation. Knockdown of P300 in both FIL‐ASPCs and Im‐FIL‐ASPCs reduced lipid accumulation and suppressed expression of adipogenic markers during differentiation (Figure S10C–E). Overexpression of P300 in Im‐FIL‐ASPCs partially rescued the impaired adipogenesis induced by PPT1 overexpression; this rescue was abolished when cells were co‐treated with the P300 inhibitor CCS1477 (Figure S10F). Conversely, ectopic P300 attenuated the anti‐adipogenic effect of 2‐BP (Figure S10G). These data indicated P300 as a downstream effector through which PPT1 modulated adipogenesis in FIL.

**FIGURE 7:**
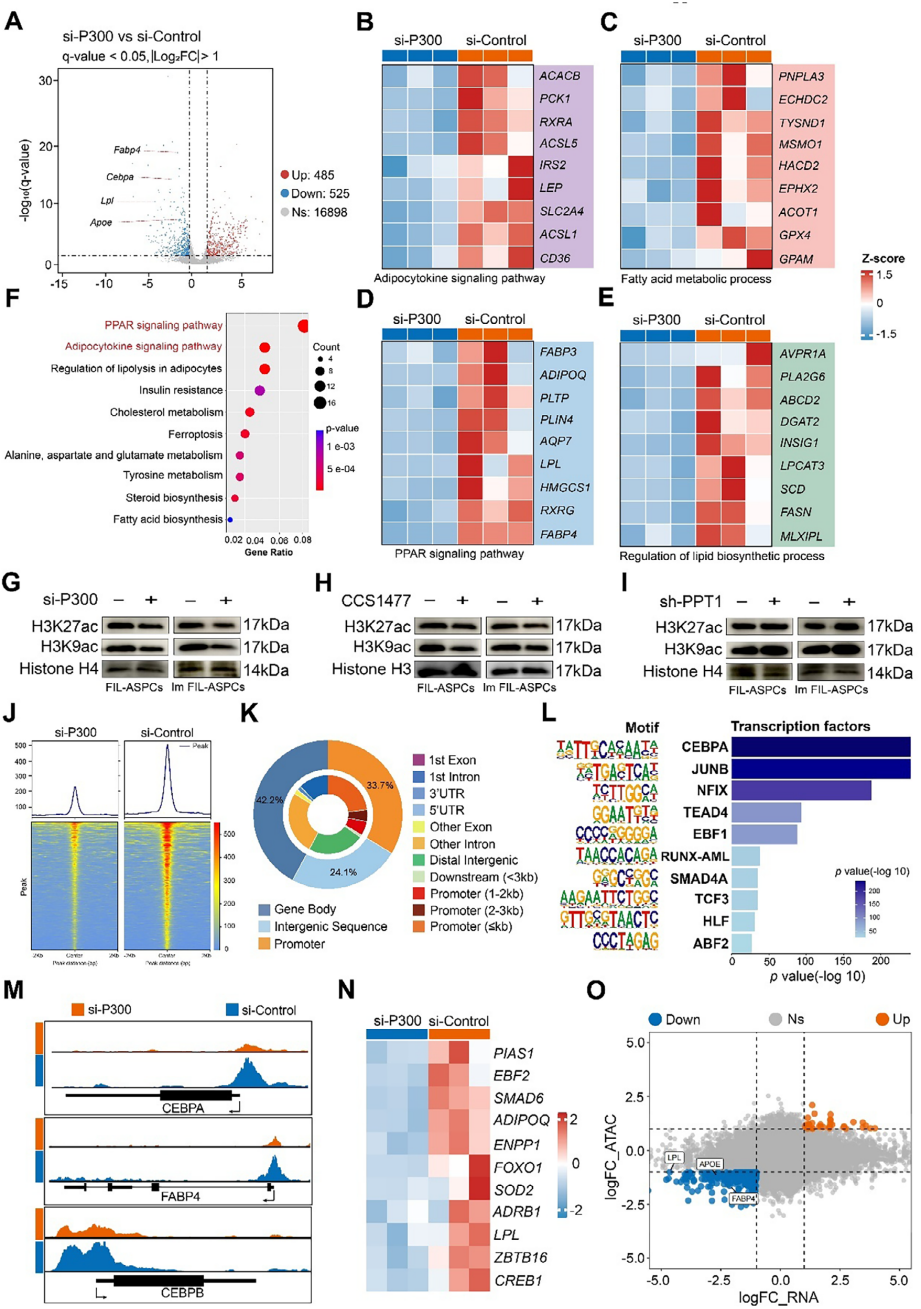
P300 promoted adipogenesis by transcriptional reprogramming. (A) Volcano plot of significantly upregulated (red) and downregulated (blue) genes identified by comparing si‐P300 or si‐Control treated FIL‐ASPCs. (B) Heatmaps showed the RNA‐seq results of differentially expressed genes related to the adipocytokine signaling pathway in si‐P300 or si‐Control treated FIL‐ASPCs. (C) Heatmaps showed the RNA‐seq results of differentially expressed genes related to the fatty acid metabolic process in si‐P300 or si‐Control treated FIL‐ASPCs. (D) Heatmaps showed the RNA‐seq results of differentially expressed genes related to the PPAR signaling pathway. (E) Heatmaps showed the RNA‐seq results of differentially expressed genes related to the regulation of lipid biosynthetic process. (F) Dot plot shows the significant Kyoto Encyclopedia of Genes and Genomes pathways among the differentially expressed genes. (G) Western blot analysis for the expression of H3K27ac and H3K9ac in FIL‐ASPCs and Im FIL‐ASPCs with or without PPT1 knockdown. (H) Western blot analysis for the expression of H3K27ac and H3K9ac in FIL‐ASPCs and Im FIL‐ASPCs treated with CCS1477. (I) Western blot analysis for the expression of H3K27ac and H3K9ac in FIL‐ASPCs and Im FIL‐ASPCs with or without PPT1 knockdown. (J) Heatmaps for differentially enriched ATAC‐seq peaks in FIL‐ASPCs with or without P300 knockdown. (K) Pie chart showed the genomic distribution of accessible regions. (L) DNA‐binding motifs enriched in the open chromatin regions of FIL‐ASPCs with or without P300 knockdown. (M) IGV maps showed the increased ATAC‐seq peaks of representative adipogenic genes. (N) Heatmaps showed the ATAC‐seq analysis of upregulated genes related to PPAR signaling pathway. (O) Quadrantal diagram depicting the overlap of significantly differentially expressed genes from bulk RNA‐seq and genes with significantly variational peaks from ATAC‐seq data. Both upregulated (orange); both downregulated (blue).

To dissect how P300 regulated adipogenesis, we performed RNA‐seq on FIL‐ASPCs undergoing adipogenic induction after P300 knockdown. P300 knockdown evoked a genome‐wide transcriptional shift: 485 genes were upregulated and 525 downregulated (FDR < 0.05, |log_2_FC| ≥ 1), indicating that P300 remodeled the transcriptional program on a global scale. Consistent with the results of the phenotypic examinations, the genes whose expression was downregulated in the P300‐knockdown FIL‐ASPCs, such as Fabp4, Lpl, and Fasn, were related to the PPAR signaling pathway and regulation of lipid biosynthetic process. Moreover, the expression of markers of adipogenesis, such as cebpa, was downregulated (Figure 7A–E). Kyoto encyclopedia of genes and genomes (KEGG) pathway analysis further revealed that differentially expressed genes were concentrated in multiple pathways previously linked to adipocyte differentiation (Figure 7F).

The HAT domain of P300 catalyzes the transfer of an acetyl group from acetyl‐CoA to lysine residues on histones, which neutralizes their positive charge, weakens the interaction between histones and DNA, and promotes chromatin relaxation, thereby facilitating the binding of transcription factors and RNA polymerase [34]. Among various modification sites, H3K27ac and H3K9ac are frequently regulated by P300 [35, 36, 37]. Knockdown of P300 or treatment with CSS1477 reduced the global levels of H3K9ac and H3K27ac in FIL‐ASPCs and Im FIL‐ASPCs (Figure 7G–H). In contrast, knockdown of PPT1 led to an increase in both modifications, which can be attributed to the antagonistic effect of P300 palmitoylation on lysosomal degradation, thereby maintaining its protein stability (Figure 7I). We then tested several P300 inhibitors and observed a dose‐dependent reduction in the levels of H3K27ac and H3K9ac (Figure S11A–C). Chip‐qPCR analysis confirmed that P300 knockdown reduced the enrichment of H3K27ac and H3K9ac at the promoter of adipogenic genes (Figure S11D,E).

To further investigate how P300 regulated the adipogenesis at the epigenetic level, we performed ATAC‐seq of the FIL‐ASPCs after P300 knockdown. Analysis of ATAC‐seq peak enrichment in regions surrounding transcription start sites (TSS ± 2 kb) across the genome revealed that knockdown of P300 significantly suppressed overall chromatin accessibility (Figure 7J). After annotating the differential peaks based on genomic distribution features, we found that the majority of enriched peaks were located in gene body and promoter regions (Figure 7K), corresponding to 740 upregulated and 5,506 downregulated annotated genes, respectively (Figure S11F). To explore the relationship between accessibility changes and specific transcription factors, we performed HOMER motif analysis on the enriched open chromatin regions. The results showed that the enrichment of CEBPA, a key transcription factor involved in adipogenic differentiation, was significantly reduced upon P300 knockdown (Figure 7L). Notably, consistent with RNA‐seq findings, ATAC‐seq profile snapshots demonstrated that the promoter regions of genes known to be associated with adipogenesis (Cebpa and Fabp4) and the PPAR signaling pathway (Adipoq and Lpl) exhibited markedly fewer accessible peaks in P300‐knockdown FIL‐ASPCs compared to control cells (Figure 7M,N). To further elucidate the mechanism by which P300 regulates adipogenesis at the transcriptional level, we integrated ATAC‐seq and RNA‐seq data. This combined analysis identified 255 overlapping differentially expressed genes (33 upregulated and 222 downregulated) common to both datasets (Figure 7O). RNA sequencing data indicated that several downregulated genes closely related to adipogenic differentiation displayed less accessible promoter regions (Figure 7O). To interpret the biological significance of chromatin accessibility changes identified via ATAC‐seq, we performed GO and KEGG pathway enrichment analyses on genes associated with differentially accessible regions. GO analysis indicated that P300 knockdown significantly affected lipid‐related functions and differentiation processes in adipocytes, while KEGG pathway analysis highlighted a pronounced impact on the PPAR signaling pathway following P300 knockdown (Figure S11G,H). These results suggested that high intracellular levels of P300 in FIL‐ASPCs promote adipogenic differentiation by enhancing chromatin openness and transcriptional expression of adipogenic genes.

### 2.7 Palmitoylation Inhibited P300 Phase Separation and Reduced Histone Acetyltransferase Activity

**FIGURE 8:**
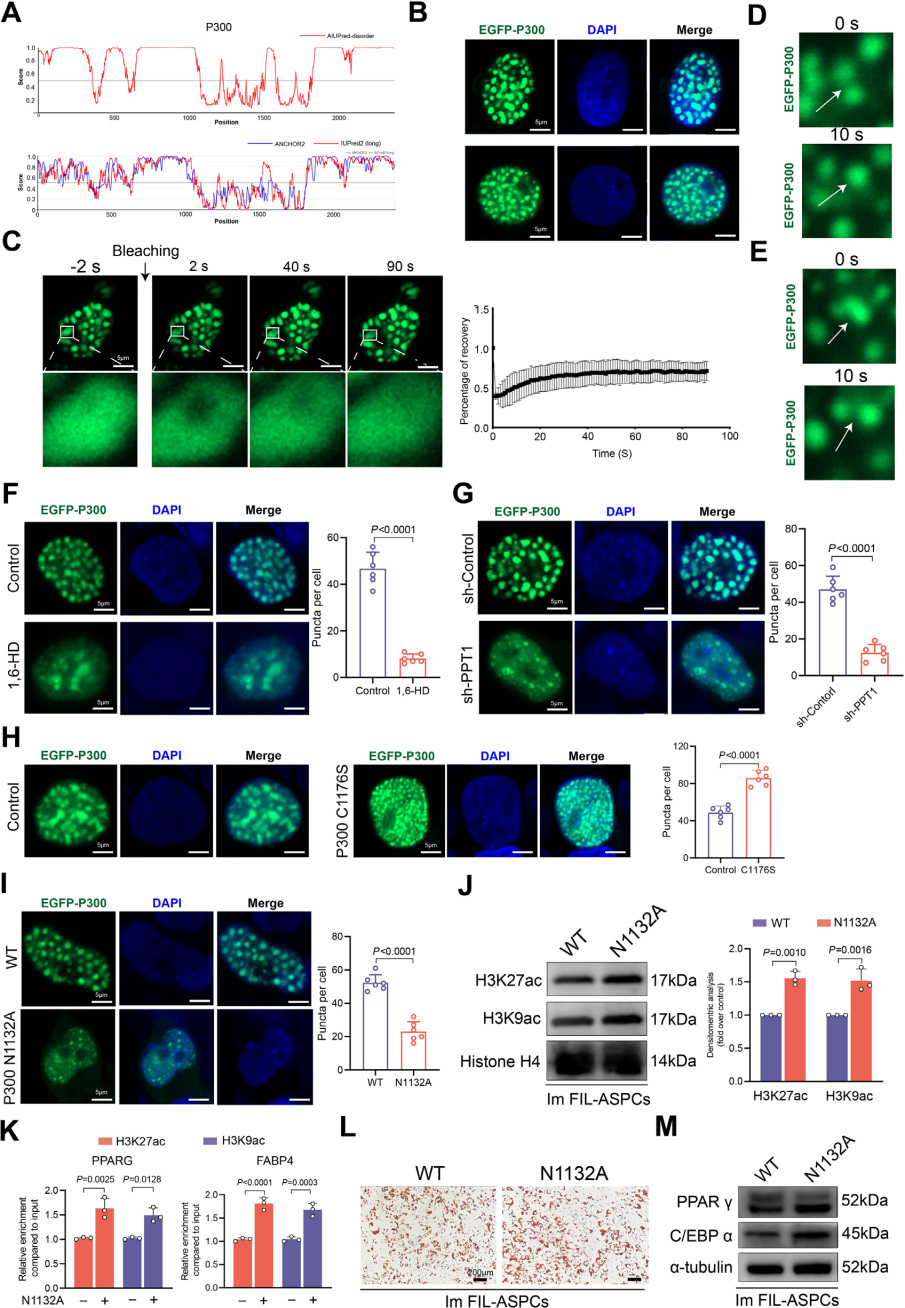
P300 palmitoylation inhibited phase separation. (A) Bioinformatics identified a major IDR in P300's amino acid sequence. (B) A droplet formation assay examined P300's cellular distribution and status. (C) FRAP experiments evaluated IGF2BP1 spot dynamics, with the right panel displaying the fluorescence recovery curve. (D) In vivo droplets coalescence of P300. (E) In vivo droplets separation of P300. (F) A droplet formation assay examined P300's cellular distribution and status in Im FIL‐ASPCs with or without 1,6‐HD treatment. (G) A droplet formation assay examined P300's cellular distribution and status in Im FIL‐ASPCs with or without PPT1 knockdown. (H) A droplet formation assay examined P300's cellular distribution and status in Im FIL‐ASPCs with or without P300 C1176S transfected. (I) A droplet formation assay examined P300's cellular distribution and status in Im FIL‐ASPCs with or without P300 N1132A transfection. (J) Western blot analysis for the expression of H3K27ac and H3K9ac in Im FIL‐ASPCs with or without P300 N1132A transfection. (K) H3K27ac and H3K9ac ChIP‐qPCR validation for selected gene promoter regions (PPARG and FABP4) in Im FIL‐ASPCs with different interventions. (L) Oil Red O staining was used to evaluate the effect of P300 N1132A on lipid droplet accumulation at day 8 of adipogenesis. Scale bar: 200 µm. (M) Western blot analysis showed that the protein levels of PPAR γ, C/EBP α, and FABP 4 in Im FIL‐ASPCs upon P300 N1132A transfection compared with the wild type at day 3 of adipogenesis. Data were analyzed by unpaired two‐sided Student's *t* tests (F, G, H, I, J, and K) and were presented as mean ± SD with at least three replicate experiments.

P300 contains large intrinsically disordered regions (Figure 8A), which are known to contribute to the formation of phase separation condensates. It has been reported to form condensates in the nucleus. Its phase separation capacity was depended on key catalytic core components, including the histone acetyltransferase (HAT) domain, the autoinhibition loop, and the bromodomain [38]. Given that the C1176 palmitoylation was located within these structural regions, we sought to investigate whether palmitoylation regulated P300 phase separation. We transfected cells with a plasmid encoding GFP‐tagged P300 and subsequently observed prominent P300 puncta formation via live‐cell imaging (Figure 8B), along with the fusion and fission dynamics of these droplets (Figure 8D,E). The dynamic recombination and fast exchange kinetics of liquid‐like condensates formed by P300 were determined by measuring the fluorescence recovery rate after photobleaching. After photobleaching, EGFP‐P300 puncta recovered fluorescence on a time‐scale of seconds (Figure 8C).

Treatment with 1,6‐hexanediol (1,6‐HD) significantly reduced the number of P300 condensates (Figure 8F). Interestingly, knockdown of PPT1 similarly led to a decrease in P300 condensates, whereas introduction of the C1176S mutant markedly increased condensate number, suggesting that palmitoylation of P300 inhibited its phase separation (Figure 8G,H). It has been reported that the formation of P300 condensates reduced its HAT activity and decreased the level of H3K27ac, while the P300 N1132A mutation inhibited P300 phase separation [38]. Given the broad‐spectrum activity of 1,6‐HD in inhibiting phase separation, we generated the P300 N1132A mutation to specifically examine the effect of suppressed phase separation on adipogenic differentiation. Consistently, the P300 N1132A mutation inhibited condensate formation (Figure 8I) but increased the overall abundance of H3K27ac and H3K9ac (Figure 8J). ChIP‐qPCR confirmed that the P300 N1132A mutation enhanced the enrichment of H3K27ac and H3K9ac at the promoter regions of PPARG and CEBPA (Figure 8K). Furthermore, the P300 N1132A mutation also resulted in increased lipid accumulation and elevated expression of key adipogenic proteins during adipogenic differentiation (Figure 8L, M). Taken together, these findings indicated that palmitoylation of P300 not only counteracted its degradation but also maintained its HAT activity by inhibiting phase separation, thereby promoting adipogenesis.

### 2.8 P300 Mediated the Adipogenesis‐Suppressing Role of PPT1

**FIGURE 9:**
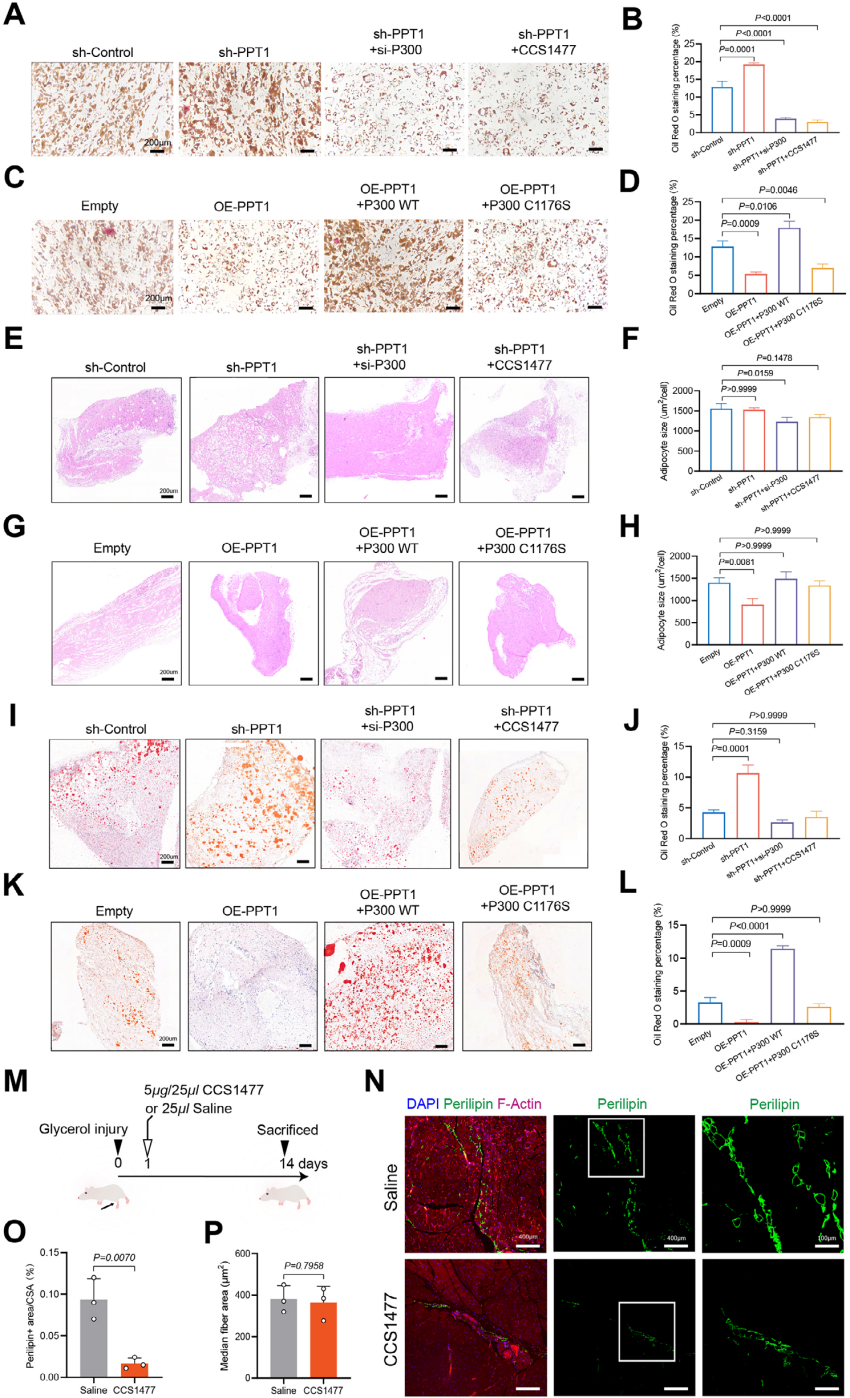
PPT1‐P300 axis regulated the adipogenesis and adipocyte infiltration. (A, B) Oil Red O staining was used to evaluate the effect of PPT‐knockdown and P300‐knockdown on lipid droplet accumulation at day 8 of adipogenesis. Scale bar: 200 µm. (C, D) Oil Red O staining was used to evaluate the effect of PPT‐overexpression and P300‐ overexpression on lipid droplet accumulation at day 8 of adipogenesis. Scale bar: 200 µm. (E–H) H&E staining of Matrigel implants collected on day 28 and quantification of adipocytes size. Scale bar: 200 µm. (I–L) Oil Red O staining of Matrigel implants and quantification of Oil red O staining area. Scale bar: 200 µm. (M) Experimental timeline outlining in vivo glycerol injection, CCS1477 administration, and analyses. (N) Representative cross‐sections of glycerol‐injured TA muscles treated with CCS1477 or saline. Scale bars: 400 µm. (O) Perilipin area normalized by the TA cross‐sectional area. (P) Median fiber cross‐sectional area. Data were analyzed by one‐way ANOVA (B, D, F, H, J, and L) and were presented as mean ± SD with at least three replicate experiments.

**FIGURE 10:**
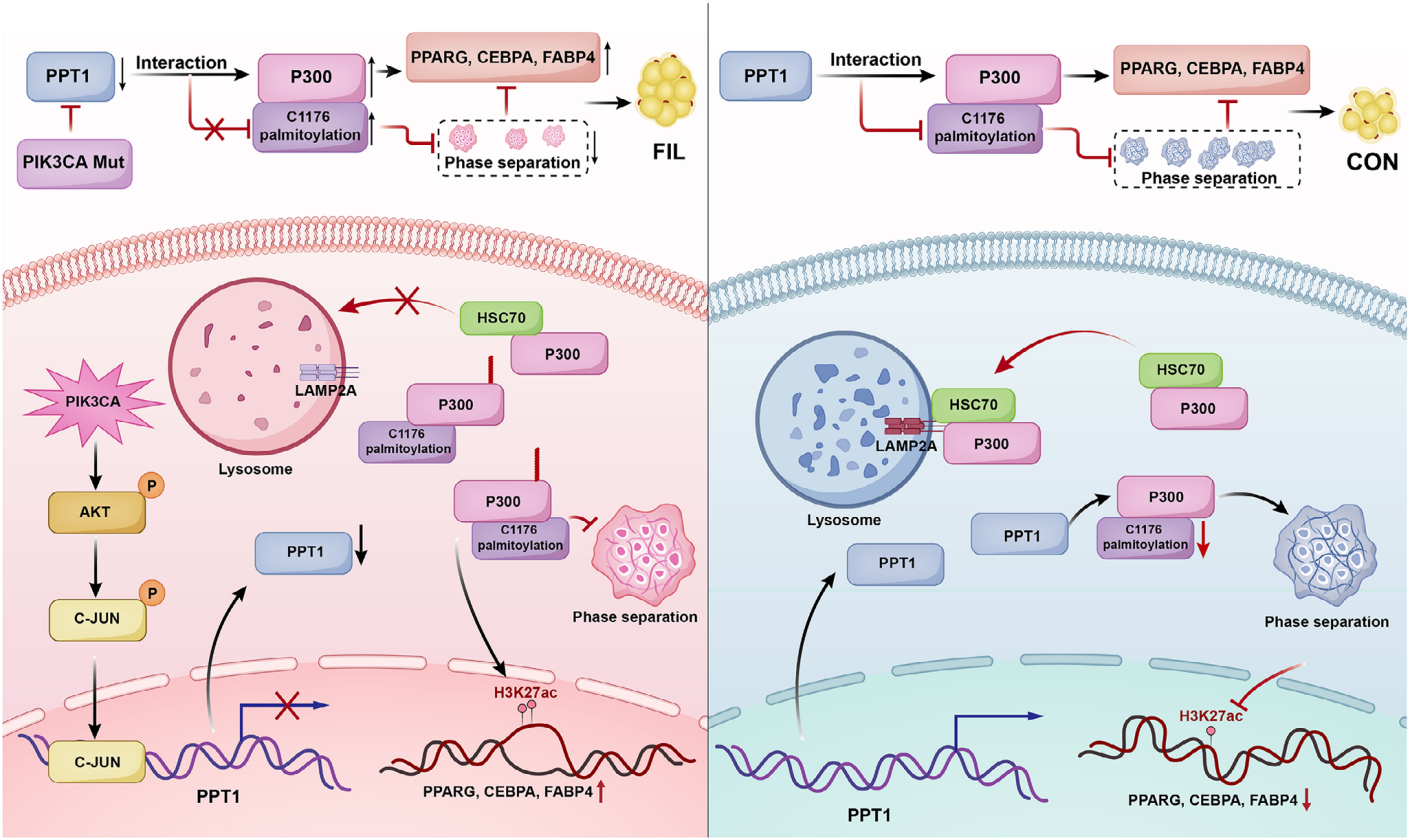
PPT1‐mediated depalmitoylation of P300 suppressed adipogenesis via promoting phase separation condensates formation. In FIL‐ASPCs, a gain‐of‐function PIK3CA mutation hyper‐activates the PI3K–AKT signaling cascade. This signaling input recruits c‐JUN to the transcriptional start site of PPT1, resulting in robust transcriptional repression of PPT1. PPT1 downregulation increases palmitoylation of P300 at C1176, which dampens its interaction with HSC70 and subsequent lysosomal degradation. Moreover, C1176‐palmitoylation interferes with P300 phase‐separation condensate formation, preserving histone acetyltransferase (HAT) activity. Consequently, H3K27ac deposition at adipogenic gene promoters is elevated, driving their transcription (left). In CON‐ASPCs, PPT1 removes the C1176‐palmitoyl mark from P300, thereby decreasing P300 abundance, promoting its phase‐separated condensate formation, and reducing histone acetyltransferase activity, which collectively downregulates adipogenic gene expression (right).

To further elucidate the function of the PPT1‐P300 axis in FIL progression, PPT1‐knockdown Im FIL‐ASPCs were knocked down for P300 or treated with CCS1477. Oil O red staining indicated that the knockdown of P300 and the treatment with CCS1477 impaired the adipogenic effect of PPT1‐knockdown in Im FIL‐ASPCs (Figure 9A, B). On the other hand, overexpression of P300 partially rescued the inhibitory effect on adipogenic differentiation induced by PPT1 overexpression, whereas overexpression of P300 C1176S did not exert this effect (Figure 9C, D). Implant lipoma models showed that the group with P300 knockdown or treatment with CCS1477 inhibited adipocytes formation and lipid accumulation compared with xenografts with PPT1 knockdown alone (Figure 9E, F, I, J). Moreover, P300‐WT rescued adipogenesis inhibited by PPT1 overexpression, while P300 C1176S failed to promote adipogenesis compared with P300‐WT in PPT1 overexpressed Im FIL‐ASPCs (Figure 9G, H, K, L). Furthermore, we try to assess the efficacy of CCS1477 in suppressing intramuscular fatty infiltration in vivo using the glycerol injury model to mimick FIL phenotype. Intramuscular injection of glycerol resulted a robust and reproducible fatty infiltration without affecting muscle regeneration, thus serving as an appropriable in vivo model to assess therapeutic efficacy for improving intramuscular adipogenesis [39]. To induce injury, tibialis anterior (TA) muscles were injected with glycerol (50% v/v). CCS1477 or saline was injected into the belly of the injured TA muscles 1‐day post‐injury. The muscles were then harvested 14 days post‐injury for analyses (Figure 9M). Glycerol‐injured TAs with saline treatment exhibited a severe adipose infiltration, marked by perilipin expression within the interstitial space. However, glycerol‐injured TAs with CCS1477 treatment exhibited a significantly reduced perilipin expression (Figure 9N, O). We observed no statistically significant differences in the myofiber area distribution (Figure 9P). Taken together, all these results indicated that PPT1 interacted with P300 to increase the depalmitoylation, degradation and phase separation of P300, leading to the transcriptional inhibition of the adipogenic genes. PIK3CA mutation promoted adipogenesis in FIL by suppressing PPT1 expression and reversing the aforementioned effects (Figure 10).

## 3 Discussion

PPT1 had been extensively studied for its role in neuronal ceroid lipofuscinosis, where its loss‐of‐function led to pathological accumulation of palmitoylated proteins [40, 41]. Beyond this hereditary disorder, emerging evidence implicated PPT1 as a broader regulator of cellular homeostasis, influencing processes from autophagy and metabolism to immune responses [42, 43]. Notably, its function in mesenchymal cell differentiation, particularly adipogenesis, remains largely unexplored. Our research systematically elucidated the molecular mechanism by which *PIK3CA* activating mutations drive aberrant adipogenesis in FIL through transcriptional repression of PPT1 via the PI3K‐AKT‐c‐JUN signaling axis, subsequently modulating P300 palmitoylation and LLPS. We have, for the first time, revealed the crucial protective role of PPT1 in human benign adipose overgrowth disorders and proposed a novel regulatory paradigm‐the “palmitoylation‐phase separation‐epigenetic regulation” axis‐in determining cell differentiation. This discovery not only deepened the understanding of the pathogenesis of PROS but also provided a new theoretical foundation for developing therapeutic strategies targeting downstream effectors of PI3K signaling.

Our findings demonstrated that PIK3CA mutation activated c‐JUN, which directly bind to and repressed the PPT1 promoter, leading to transcriptional downregulation of PPT1, underscoring the crucial role of transcriptional regulation in PI3K signaling‐mediated metabolic reprogramming. Although c‐JUN can bind to the PPT1 promoter region across multiple species, its regulatory effect on PPT1 in different cell lineage required further validation. As a key mediator of stress and proliferative responses, the activation of c‐JUN in the context of PIK3CA mutation highlighted the intimate coupling between oncogenic signaling and cellular metabolic states, while also providing new insights into the pathogenesis of other PI3K‐driven disorders.

Palmitoylation significantly influenced the regulation of protein degradation pathways and kinetics. ZDHHC3‐mediated palmitoylation of B7‐H4 shielded it from lysosomal degradation, thereby sustaining tumor immune evasion [31]. Similarly, ZDHHC20‐mediated palmitoylation of YTHDF3 reduced its lysosomal degradation and promoted tumor progression [44]. Beyond lysosomal regulation, palmitoylation could also modulate proteasomal degradation. For instance, ZDHHC20‐mediated FASN palmitoylation competed with the ubiquitin‐proteasome pathway by interfering with the E3 ubiquitin ligase complex SNX8‐TRIM28 [45]. We found that downregulation of PPT1 increased P300 C1176 palmitoylation level, which in turn attenuated its lysosomal degradation via the chaperone‐mediated pathway involving HSC70. This finding functionally linked protein palmitoylation with the regulation of protein stability, thereby expanding the functional repertoire of palmitoylation in cellular signal transduction. While emerging evidence suggested that palmitoylation modulates protein function by influencing conformation, interaction networks, and subcellular localization [46], our work further revealed its role in controlling protein turnover, offering new clues for understanding cross‐talk among PTM.

Notably, we discovered that C1176 palmitoylation inhibited P300 from undergoing LLPS, thereby preserving its HAT activity and facilitating sustained transcription of adipogenic genes. As an emerging frontier in cell biology, phase separation has been widely implicated in transcriptional regulation, chromatin organization, and cellular differentiation. To date, only a limited number of studies have addressed the regulatory role of palmitoylation in phase separation, while other PTMs have been reported to modulate condensate formation. For example, PARP1‐mediated ADP‐ribosylation disrupts the phase separation of cyclin T1, a key subunit of the transcription elongation factor P‐TEFb, thereby suppressing global gene transcription [47]. Additionally, disruption of ERCC6L2 phase separation rendered CtIP susceptible to RNF138‐mediated ubiquitination and degradation, indicating that phase separation can also regulate protein PTMs [48]. Our study provided the additional evidence linking palmitoylation with the phase separation behavior of a transcriptional coactivator, suggesting that lipid modification may modulate protein function by altering their condensation properties. This offers a novel molecular basis for understanding the formation and regulation of biomolecular condensates in cells.

In addition to FIL, other benign lipomatous disorders such as multiple symmetrical lipomatosis (MSL) also exhibit mosaic‐like adipocyte hyperplasia, albeit with distinct clinical and molecular profiles [49]. The pathogenesis of MSL is being dissected at the molecular–genetic level from several complementary angles. Adipose tissue from MSL patients shows constitutive overactivation of AKT, CK2 and ERK1/2. Compared with controls, MSL‐derived adipose stem cells are more numerous, proliferate faster, and display superior clonogenicity and adipogenic capacity, indicating a heightened white‐adipogenic potential [50]. CAPSL, a recently recognized regulator of adipocyte biology, is consistently downregulated in MSL; the resulting autophagic imbalance may constitute an additional disease‐driving mechanism. Concordant with CAPSL suppression, UCP1 is upregulated in the same tissue [51]. At the mitochondrial level, a subset of patients carry either the classic m.8344A>G or a novel m.8357T>C mutation in the mt‐tRNALys gene [52, 53]. Germ‐line mutations in CBLB (c.197A>T), MFN2 (c.2119C>T) and biallelic loss‐of‐function variants in LIPE have also been validated: they blunt the IRS1‐PI3K‐AKT axis, derange mitochondrial fusion, or remove hormone‐sensitive lipase, respectively, thereby uncoupling adipocyte differentiation from lipolysis [49]. Collectively, MSL pathobiology emerges from the intersection of metabolic, genetic and micro‐environmental cues. By contrast, FIL lacks an equivalent body of systematic research. The two disorders nevertheless appear to share pathways related to adipocyte origin, stem‐cell behavior and energy‐metabolism dysfunction, making them attractive candidates for comparative studies.

From a pathophysiological perspective, this study established a complete signaling pathway from somatic mutation to tissue overgrowth, identifying potential targets for precision therapy in PROS. While current research on PI3K pathway inhibitors has primarily focused on malignancies, our data suggested that targeting downstream effectors such as PPT1 or P300 may enable safer and more controlled management of localized tissue overgrowth with slightly side effects. Furthermore, the dynamic balance of palmitoylation and depalmitoylation represented a promising druggable target with broad translational potential.

Nevertheless, our study has certain limitations. For instance, the structural basis by which P300 palmitoylation influenced its phase separation remained unclear, and its generalizability across other cell types or disease models required further investigation. Additionally, whether other PPT1 substrates contribute to FIL pathogenesis, and whether different PIK3CA mutant location led to heterogeneous signaling outputs, represented important directions for future research. Building upon this work, future studies should aim to establish more disease‐relevant models (conditional knock‐in mice harboring the hotspot PIK3CA mutations) to systematically compare the efficacy and safety of PIK3CA inhibitors versus strategies targeting the downstream PPT1/P300 axis. Furthermore, investigating whether the palmitoylation‐phase separation‐epigenetic axis identified here represents a common mechanism in other PIK3CA‐driven overgrowth disorders or even in metabolic diseases characterized by aberrant adipogenesis will be of great interest.

## 4 Conclusion

In summary, this study defined a pathogenic signaling cascade in FIL, linking PIK3CA mutation to suppressed PPT1 expression and promoted P300 palmitoylation. This key modification stabilized P300 and inhibited its phase separation, ultimately enabling a pro‐adipogenic transcriptional program.

## 5 Methods and Materials

### 5.1 Human Subject and Ethics Approval

The patients’ clinical characteristics were presented in Table S1. Adipose tissues from 20 FIL patients and 13 CON patients were collected for experimental analyses. This study was conducted in accordance with the Declaration of Helsinki and approved by the Ethics Committee of Shanghai Ninth People's Hospital. The approval number was No.SH9H‐2022‐T215‐1. ASPCs were isolated and collected from facial adipose tissue, with written consent of donors.

### 5.2 ASPCs Isolation, Culture, and Identification

Detailed protocol can be found in our previous publication [54].

### 5.3 Adipogenic Induction

In short, we used the Cyagen adipogenic induction kit (Cyagen Biosciences, HUXMD‐90031); the detailed protocol can be found in our previous publication. When required, Induction solution A and B were supplemented with 2‐BP (MedChemExpress, HY‐111770), BYL‐719 (MedChemExpress, HY‐15244), T‐5224 (MedChemExpress, HY‐12270) or CCS1477 (MedChemExpress, HY‐111784). Cells were harvested on day 4 or 8 for subsequent experiments.

### 5.4 Oil Red O Staining

Oil Red O staining was performed according to the instructions of the Modified Oil Red O Staining Kit (Beyotime, C0158S). Quantitative analysis of Oil red O staining area was determined using Image‐Pro Plus 6.0 software.

### 5.5 Mice

All animal procedures were conducted under the approved protocol by the Committee of Animal Care and Use for Research and Education of Shanghai Jiao Tong University School of Medicine. Four weeks old BALB/C nude mice and 8 weeks old C57BL/6J mice were purchased from the Shanghai SLAC Laboratory Animal Co. Both male and female mice were used in a randomized manner. These mice were then individually housed under a 12‐hour light/dark cycle, with access to high‐fat or basic diet and water.

### 5.6 Implant Lipoma Experiment

A total of 1 × 10^6^ ASPCs were resuspended in 50 µL DPBS and blended 1:1 with Matrigel (Corning) on ice. The 100 µL cell–Matrigel mixture (1 × 10^6^ cells per mouse) was injected subcutaneously into the dorsal flank of BALB/c nude mice (*n* = 5 per group). Four weeks after implantation, animals were euthanized by cervical dislocation and the xenografts were collected for histological analysis.

### 5.7 Adipose Infiltration Model

8‐week‐old C57BL/6J mice were anesthetized with isoflurane. 50 µL 50% glycerol in saline was intramuscularly injected into TA muscles. After 24 h, 5 µg/25 µL CCS1477 or 25 µL saline were injected into the TA muscles in a randomized manner. Buprenorphine shots were subcutaneously administered every 12 h at the onset of the procedure for 3 days. Mice were sacrificed on day 14 for histological analyses.

### 5.8 AAV8‐Mediated Selective PPT1 Overexpression in Adipocytes

AAV8‐FABP4‐PPT1‐eGFP or AAV8‐FABP4‐Scramble‐eGFP were procured from Genomeditech. A dose of 5×10^11^ virus was resuspended in 150 µL sterile PBS. Two‐month‐old male C57BL/6J mice were anesthetized with isoflurane, after which the viral suspension was delivered by lateral‐tail‐vein injection. Six weeks later, PPT1 overexpression efficiency was evaluated by immunofluorescence and Western blot.

### 5.9 Western Blot

Harvested adipose or ASPCs samples were homogenized and lysed in RIPA lysis buffer containing protease (Epizyme, GRF101) and phosphatase inhibitor cocktails (Epizyme, GRF102) on ice. The protein concentration was measured via a BCA protein assay kit (Epizyme, ZJ102). Total protein was separated via 10% SDS‐PAGE and electrophoretically transferred onto PVDF membranes (Millipore). After nonspecific background staining was blocked with Protein free rapid blocking buffer (1×) (Epizyme, PS108P) at room temperature for 10 min, the membranes were incubated with primary antibodies at 4°C overnight. The next day, the membranes were washed with Tris‐buffered saline containing 0.1% Tween 20 and incubated with an isotype‐matched secondary antibody for 45 min at room temperature. The primary antibodies used were as follows: anti‐PPT1 (Proteintech, 29653‐1‐AP), anti‐p110α (Proteintech, 67071‐1‐Ig), anti‐AKT (Cell Signaling Technology, 4691), anti‐phospho‐AKT (Cell Signaling Technology, 9271), anti‐JUN (Proteintech, 24909‐1‐AP), and anti‐phospho‐JUN (Proteintech, 28891‐1‐AP), anti‐PPAR γ (Cell Signaling Technology, 2443S), anti‐C/EBP α (Proteintech, 13274‐1‐AP), anti‐FABP 4 (Proteintech, 12802‐1‐AP), anti‐P300 (Proteintech, 20695‐1‐AP) anti‐H3K9ac (HUABIO, HA722132), anti‐H3K27ac (HUABIO, HA500046), anti‐Histone H4 (HUABIO, ET1612‐43), anti‐Flag (MERCK, F7425), anti‐Myc (Proteintech, 60003‐2‐Ig), anti‐VPS4A (Epizyme, 95M49L94), anti‐p62 (Epizyme, 78L28M94). The blot signals were visualized using Pierce ECL Western blot Substrate (Thermo Fisher Scientific, 32209). The abundance of each target protein was quantified via ImageJ software (NIH).

### 5.10 RNA Isolation, Reverse Transcription, and Real‐Time Quantitative PCR (qPCR)

Total RNA was extracted from cultured cells using TRIzol Reagent (Invitrogen, USA), and cDNA was synthesized using PrimeScript RT Reagent Mix (Takara Bio, USA) according to the manufacturer's instructions. qPCR was performed using SYBR Green PCR Master Mix (Life Technologies, USA) and an ABI 7500 real‐time PCR system (Applied Biosystems, USA). The levels of targets were normalized to those of ACTB and to those of control samples. The primers for qPCR are listed in Table S2.

### 5.11 LC‐MS/MS

For proteomic profiling, peptides were analyzed using an OE480 Hybrid Quadrupole‐Orbitrap MS (Thermo Fisher Scientific) connected to an EASY‐nLC 1200 UHPLC system (Thermo Fisher Scientific). Lyophilized peptides were reconstituted in Solvent A (0.1% formic acid in water), loaded onto a 2 cm, 100 µm‐i.d. trap column (Dr. Maisch GmbH) at the same solvent, and subsequently separated on a 15 cm×150 µm‐i.d. analytical column packed in‐house with 1.9 µm ReproSil‐Pur C18‐AQ beads (Dr. Maisch GmbH). A 75‐min gradient delivered at 600 nL min^−^^1^ was applied: 0–10 min, 4–15% B; 10–60 min, 15–30% B; 60–69 min, 30–50% B; 69–70 min, 50–100% B; 70–75 min, 100% B (Solvent B: 0.1% formic acid in 80% ACN). Eluting peptides were electrosprayed at 2.4 kV and transferred into the mass spectrometer. Full‐scan MS1 spectra (m/z 300–1,400) were acquired in the Orbitrap at 120,000 resolution with an AGC target of 3 × 10^6^ and a maximum injection time of 80 ms. Data‐dependent acquisition used a 1‐s cycle time; precursors were isolated and fragmented by HCD (NCE 30%). MS2 spectra were recorded in the Orbitrap at 7,500 resolution, AGC 5 × 10^4^, and 22 ms maximum injection time. Selected ions were excluded from repeated fragmentation for 12 s. FAIMS compensation voltages were set to –45 V and –65 V. Raw files were processed with Proteome Discoverer 2.3 for peptide identification and quantification.

### 5.12 Acyl‐Biotin‐Exchange (ABE) Assay

To assess P300 S‐palmitoylation, an acyl‐biotin exchange (ABE) protocol was employed. Treated cells were harvested in ice‐cold lysis buffer (50 mM HEPES pH 7.4, 150 mM NaCl, 5 mM EDTA, 1% Triton X‐100, 1×Halt protease/phosphatase inhibitor) for 30 min. Proteins were recovered by chloroform/methanol precipitation (1:3:2 v/v/v lysate:methanol:CHCl_3_) and the resulting pellet was solubilized in 4% SDS buffer. After five‐fold dilution into fresh lysis buffer supplemented with 25 mM N‐ethylmaleimide (NEM; Sigma E3876), samples were rotated overnight at 4°C to block free thiols. Residual NEM was eliminated by three successive CM precipitations, followed by resuspension in 300 µL 4SB. Each specimen was then split into two equal aliquots. The experimental fraction was diluted 1:5 into HAM buffer (0.2% Triton X‐100, 1× inhibitors, 1 M hydroxylamine and 1 mM EZ‐link HPDP‐biotin to cleave thioester‐linked palmitates and label newly exposed cysteines with biotin. The control aliquot received identical buffer except that hydroxylamine was replaced by 50 mM Tris‐HCl pH 7.4, leaving palmitoyl groups intact and preventing biotin incorporation. Both reactions were incubated at room temperature for 1 h with gentle mixing. Unincorporated biotin was subsequently removed by three rounds of CM precipitation, and pellets were dissolved in 4SB, then diluted 10‐fold into Tris‐based lysis buffer. Biotinylated proteins were captured with streptavidin beads, thoroughly washed, eluted in SDS‐PAGE sample buffer and analyzed by immunoblotting.

### 5.13 Click‐iT Identification of P300 Palmitoylation

FIL‐ASPCs were exposed to 100 mM Click‐iT palmitic acid azide for 6 h. Following labeling, cells were harvested and lysed to obtain total protein. Click‐iT Protein Reaction Buffer Kit (Thermo, C10276) was used to catalyze the reaction of protein samples with biotin‐alkyne. Biotin alkyne azide–palmitic‐protein complex was precipitated with streptavidin, and samples were immunoblotted for P300.

### 5.14 Chromatin Immunoprecipitation and Quantitative PCR Validation (ChIP‐qPCR)

FIL‐ASPCs were cross‐linked with 1% formaldehyde for 15 min at room temperature, then the reaction was quenched by adding glycine to 0.125 M. Cells were lysed and nuclei released by Dounce homogenization in ice‐cold lysis buffer. Chromatin was sheared to 300–500 bp fragments by sonication. An input sample was generated by digesting an aliquot of sheared chromatin with RNase A and proteinase K, reversing cross‐links at 65°C overnight, and purifying DNA by ethanol precipitation; concentration was determined with a NanoDrop spectrophotometer and used to calculate total chromatin recovery. For immunoprecipitation, 30 µg of chromatin was pre‐cleared with protein A‐agarose beads (Invitrogen) and then incubated with anti‐JUN, anti‐H3K9ac and anti‐H3K27ac antibody. Immune complexes were washed, eluted in SDS‐containing buffer, treated with RNase and proteinase K, and cross‐links were reversed (65°C, overnight). ChIP DNA was recovered by phenol–chloroform extraction followed by ethanol precipitation. Enrichment was verified by triplicate qPCR with SYBR Green Supermix (Bio‐Rad) on selected genomic loci; values were normalized to reactions performed on matching input DNA. Primer sequences are listed in Table S2.

### 5.15 Proximity Ligation Assay (PLA)

FIL‐ASPCs were plated on confocal‐grade dishes and left to attach overnight. After 24 h, the monolayers were fixed in 4% paraformaldehyde for 1 h at 37°C, then permeabilized and blocked. Primary antibodies directed against PPT1 and P300 were applied overnight at 4°C On the following day the NavinciFlex Cell MR protocol (Navinci, Atto647N) was followed for probe hybridization, ligation and rolling‐circle amplification. Nuclei were counterstained with DAPI and proximity‐ligation signals were captured on a Thunder Imager high‐speed inverted fluorescence microscope (Leica).

### 5.16 Isolation of Lysosomal Proteins

Lysosomal fractions were isolated according to the kit manual (Thermo). In brief, 5 × 10^7^ pancreatic cancer cells were detached with the supplied reagents, sonicated, and the homogenate subjected to ultracentrifugation at 145,000 × *g*, 4°C. The resulting pellet was resuspended in Laemmli buffer and analyzed by immunoblotting.

### 5.17 Plasmid and siRNA Transfection

Plasmids containing Flag‐PPT1 and Myc‐P300 were obtained from Zuorun Bio (Shanghai, China). Small silencing RNAs (si‐JUN, si‐HSC70, si‐P300) were purchased from Genomeditech Bio (Shanghai, China). Plasmids or siRNAs were transfected into 60%–70% confluent FIL‐ASPCs with Lipo8000 reagent (Beyotime, C0533). Medium was changed to basal medium after 4–6 h transfection before the following experiments.

### 5.18 Immunohistochemistry

For immunohistochemistry staining, the deparaffinized and hydrated histological slides of adipose tissue were blocked by 5% bovine serum albumin for 30 min at room temperature and incubated overnight (4°C) with primary antibodies. Next, the slides were washed with TBST (G0004; Servicebio), followed by the incubation with secondary antibodies, HRP‐conjugated goat anti‐rabbit IgG (GB23303; Servicebio).

### 5.19 Immunofluorescent Staining

Sections were first deparaffinized, followed by antigen retrieval using either EDTA (pH = 9.0) or sodium citrate (pH = 6.0) solutions, applying high pressure for 2 min. Afterward, the sections were incubated with 10% goat serum at ambient temperature for 1 h to block non‐specific binding, then treated with primary antibodies overnight at 4°C. The sections underwent three washes with Phosphate buffered saline before being incubated with Alexa Fluor 488 goat anti‐rabbit (A‐11,008, Invitrogen) and Alexa Fluor 594 goat anti‐mouse (ab150116, Abcam) secondary antibodies for 40 min at room temperature. Finally, sections were coverslipped using a 4′,6‐diamidino‐2‐phenylindole (DAPI) based mounting medium (ZLI‐9557, ZSGB‐BIO).

### 5.20 Lentivirus Packaging

We prepared lentivirus to knockdown or overexpress the expression of PPT1 and PIK3CA. The lentivirus was obtained from Zuorun Biotech. For transduction, ASPCs were incubated with virus‐containing supernatant (MOI = 10) in the presence of 10 µL polybrene. After 10 h, infected cells were selected with puromycin supernatant. The sequence for gene modulation is listed in Table S3.

### 5.21 scRNA Sequencing

RNA Sequencing and Data Analysis Experiments were performed by Genefund Biotech (Shanghai, China). In brief, adipose tissue or ASPCs were homogenized and total RNA isolated with the RNeasy Mini Kit (QIAGEN, 74 004). Concentration was determined on a NanoDrop 2000 spectrophotometer and integrity verified on an Agilent 2100 Bioanalyzer; only samples with RIN ≥ 8.0 were retained. For each specimen, 3 µg of RNA was converted into an Illumina‐compatible library using the VAHTS Universal V6 RNA‐seq Library Prep Kit following the vendor's protocol. Paired‐end 150‐bp reads were generated on a NovaSeq 6000 instrument. Gene‐level abundances were calculated as FPKM values by featureCounts (v2.0.1). Differential expression was assessed with DESeq2 (v1.30.1); genes exhibiting an adjusted *p*‐value < 0.05 and |log2 fold‐change| > 1 (or > 0.5 where stated) were retained for downstream analyses. Volcano plots were drawn with the ggplot2 R package, and heat‐maps were generated in GENE‐E (Broad Institute).The single‐cell analysis was performed by Singleron Biotechnologies, sourced from our prior sequencing data deposited in the GEO database under accession number: GSE267777. The data underwent processing using the Seurat package (version 4.0.3) within the R‐studio environment (version 4.0.2), converting them into Seurat object format, and quality control measures were implemented for each cell‐gene matrix of the samples. Subsequently, a comprehensive analysis was conducted across all samples to pinpoint 2000 genes exhibiting high variability, which were then employed to consolidate the matrices into a unified Seurat object. This process also involved the mitigation of batch effects and the reduction of data dimensionality through principal component analysis (PCA). The resulting cell clusters were depicted graphically using Uniform Manifold Approximation and Projection (UMAP), and cell types were classified based on established marker genes.

Experiments were performed by OE Biotech (Shanghai, China). In brief, adipose tissue or ASPCs were homogenized and total RNA isolated with the RNeasy Mini Kit (QIAGEN, 74 004). Concentration was determined on a NanoDrop 2000 spectrophotometer and integrity verified on an Agilent 2100 Bioanalyzer; only samples with RIN ≥ 8.0 were retained. For each specimen, 3 µg of RNA was converted into an Illumina‐compatible library using the VAHTS Universal V6 RNA‐seq Library Prep Kit following the vendor's protocol. Paired‐end 150‐bp reads were generated on a NovaSeq 6000 instrument. Gene‐level abundances were calculated as FPKM values by featureCounts (v2.0.1). Differential expression was assessed with DESeq2 (v1.30.1); genes exhibiting an adjusted *p*‐value < 0.05 and |log2 fold‐change| > 1 (or > 0.5 where stated) were retained for downstream analyses. Volcano plots were drawn with the ggplot2 R package, and heat‐maps were generated in GENE‐E (Broad Institute).

### 5.22 ATAC Sequencing and Data Analysis

ATAC sequencing (ATAC‐seq) was performed by Genefund Biotech (Shanghai, China). In brief, ASPCs were lysed and nuclei were isolated following a standard protocol. Transposition reactions and Illumina‐compatible DNA libraries were constructed with the TruePrep DNA Library Prep Kit V2 for Illumina (Vazyme, TD501) as recommended by the supplier. After amplification and quality control, the libraries were loaded onto a NovaSeq 6000 instrument and 150‐bp paired‐end reads were produced. Raw reads were cleaned with fastp (v0.23.1) to remove adapter contamination and low‐quality bases. Sambamba (v0.7.1) handled sam/bam conversion and duplicate marking. TSS‐centered read densities were plotted with DeepTools (v2.4.1), genomic feature distribution was surveyed with RSeQC (v2.6), motifs were discovered with Homer (v4.10), and differential peaks were called using csaw (v1.24.3).

### 5.23 Molecular‐Dynamics (MD) Simulations

All atomistic MD calculations were performed with GROMACS 2023 to quantify the conformational stability and interfacial energetics of the protein–protein complex. The initial coordinates were taken from the best docking pose and parameterized with the CHARMM36 force field. The complex was solvated in a periodic dodecahedral box of TIP3P water molecules that extended at least 1.0 nm beyond any solute atom; Na^+^ and Cl^−^ ions were added to neutralize the net charge and to reach a physiological ionic strength of 0.15 M. After steepest‐descent energy minimization to remove unfavorable contacts, the system was gradually equilibrated under NVT conditions (300 K) followed by NPT ensemble (1 bar) using the Berendsen thermostat and Parrinello–Rahman barostat. A 10‐ns production run was executed with a 2‐fs time step; bond lengths involving hydrogen atoms were constrained with the LINCS algorithm, and long‐range electrostatics were treated with the particle‐mesh Ewald (PME) method. Trajectory frames were saved every 10 ps. Periodic‐boundary artifacts were removed and the trajectory was centered on the protein complex prior to analyses. Root‐mean‐square deviation (RMSD), root‐mean‐square fluctuation (RMSF), radius of gyration (Rg), and the number of hydrogen bonds across the interface were computed to dissect the dynamic stability and underlying interaction mechanism of the complex.

### 5.24 Fluorescence Recovery After Photobleaching (FRAP)

FRAP experiments were performed on a Zeiss LSM 980 laser scanning confocal microscope equipped with an Airyscan2 detector and a 63× oil immersion objective. For EGFP‐P300 condensates, a circular region of interest (ROI) at the center of the condensate was selected for photobleaching. Initial images were acquired at low laser power (<1%) for 3‐5 frames to establish the baseline fluorescence intensity. The selected ROI was subjected to a single high‐intensity pulse using a 488 nm laser at 80% power, achieving over 70% bleaching depth. Immediately after bleaching, time‐lapse imaging was resumed at low laser power, capturing frames at 3‐s intervals for 5 min to monitor fluorescence recovery.

### 5.25 Quantification and Statistical Analysis

GraphPad Prism (v 8.0) was used for statistical analyses. Two‐tailed Student's *t* test was utilized for comparison between the two groups. One‐way analysis of variance (ANOVA) coupled with the Bonferroni's post hoc test was used for comparisons among three or more groups. Data were presented as mean ± standard deviation (SD). *p* < 0.05 was considered of statistical significance.

## Author Contributions

Hongrui Chen: Conceptualization, Methodology, Software, Investigation, Formal Analysis, Writing – Original Draft. Zening Huang, Rui Chang: Data Curation, Writing – Original Draft. Wei Gao: Visualization, Investigation. Yajing Qiu: Resources, Supervision. Bin Sun, Xiaoxi Lin: Software, Validation, Writing – Review & Editing. Chen Hua: Visualization, Writing – Review & Editing.

## Funding

This research was supported by Fundamental research program funding of Ninth People's Hospital affiliated to Shanghai Jiao Tong university School of Medicine (JYZZ241), Top Priority Research Center of Shanghai—Plastic Surgery Research Center, Shanghai (2023ZZ02023), Fundamental Research Funds for the Central Universities (No. YG2023ZD13), and Joint Funds for the innovation of Science and Technology, Fujian province (2023Y9208).

## Declarations

**Ethics approval and consent to participate**: The study was approved by the Ethics Board of Shanghai Ninth Hospital, Shanghai Jiaotong University of Medicine (SH9H‐2022‐T215‐1). Written informed consent forms were signed by patients or patients’ parents whose facial adipose sample were collected in this study. Title of the approved project: Treatment and mechanism of Pl3K/mTOR dual‐target inhibitor (WX390) on PlK3CA‐related overgrowth spectrum (PROS). Date of approval: 06, October, 2022. All animal procedures were performed following protocols approved by the Institutional Animal Care and Use Committee (IACUC) of the Shanghai Jiao Tong University School of Medicine (SH9H‐2022‐A916‐1). Title of the approved project: Etiological Research on Limb Developmental Defects and Construction of a Precision Prevention and Treatment System: Research on the Pathogenesis and Targeted Therapy of Limb Proliferative Diseases Caused by Somatic Activating Mutations. Date of approval: 11, November, 2022.

## Consent for publication

The consent for publication was acquired from patients’ parents. All authors confirm their consent for publication.

## Conflicts of Interest

The authors declare no conflict of interest.

## Supporting information




**Supplemental File 1**: advs74036‐sup‐0001‐SuppMat.docx.

**Supplemental File 2**: advs74036‐0002‐Tables.zip.

## Data Availability

The datasets used and/or analyzed during the current study are available in NCBI's Gene Expression Omnibus (GEO) or Genome Sequence Archive (Genomics, Proteomics & Bioinformatics 2025) in National Genomics Data Center (Nucleic Acids Res 2025), China National Center for Bioinformation / Beijing Institute of Genomics and are accessible through GEO Series accession number GSE300520 (RNA‐seq in Figure 1), or GSA‐Human: HRA014905 (RNA‐seq in Figure 7), GSA‐Human: HRA014913 (ATAC‐seq in Figure 7).
