# Supplementary material for: PIK3CA Mutations Downregulate PPT1 to Promote Adipogenesis by Suppressing P300 Depalmitoylation and Phase Separation
Source: Adv Sci (Weinh). 2026 Jan 29;13(19):e23139. doi: 10.1002/advs.202523139 (PMC13045223; doi:10.1002/advs.202523139)
Supplement: Supplementary file 1 — Supplemental File 1: advs74036‐sup‐0001‐SuppMat.docx. [file ADVS-13-e23139-s002.docx]

**
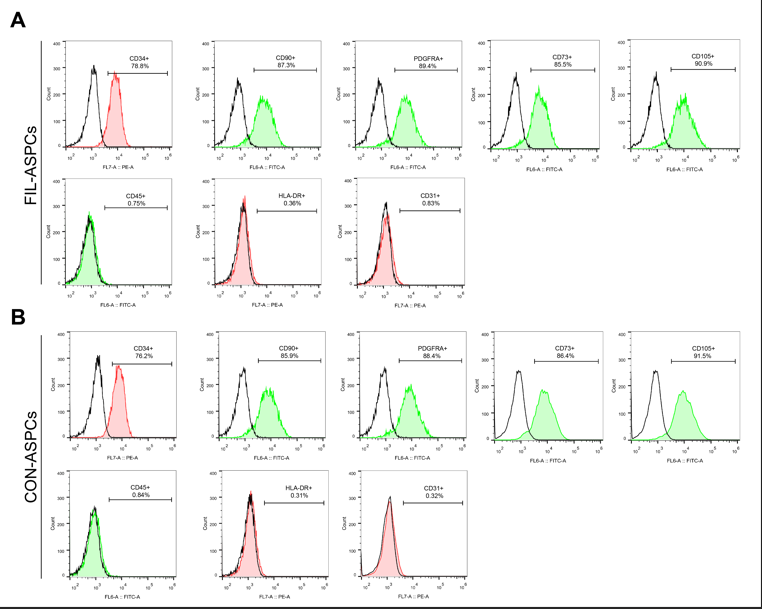
**

**Figure S1: Characterization of primary ASPCs.** The expression of the ASPCs surface markers CD34, CD90, PDGFRA, CD73 and CD105, the haematopoietic marker CD45,the immune marker HLA-DR and the endothelial marker CD31 in isolated ASPCs at passage 1 was detected by flow cytometry.


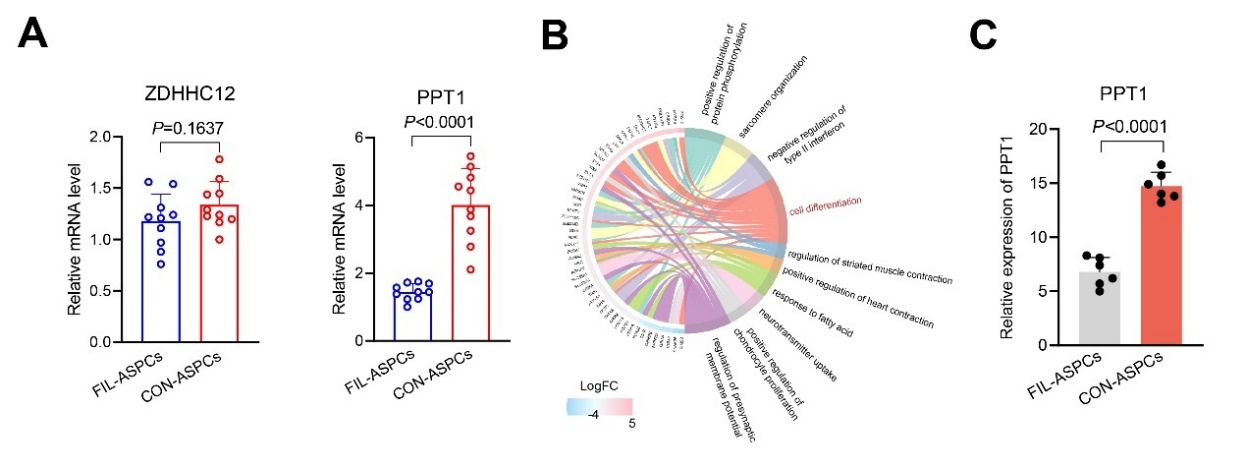


**Figure S2: Transcriptional landscape of FIL-ASPCs.** A: qPCR detected the mRNA levels of ZDHHC12 and PPT1 in FIL-ASPCs and CON-ASPCs. B: Circos analysis of differentially expressed genes. Data were analyzed by unpaired two-sided Student’s t tests (A) and were presented as mean ± SD. C: Statistical results of PPT1 levels in FIL-ASPCs and CON-ASPCs. Data were analyzed by unpaired two-sided Student’s t tests (C) and were presented as mean ± SD (n=6).


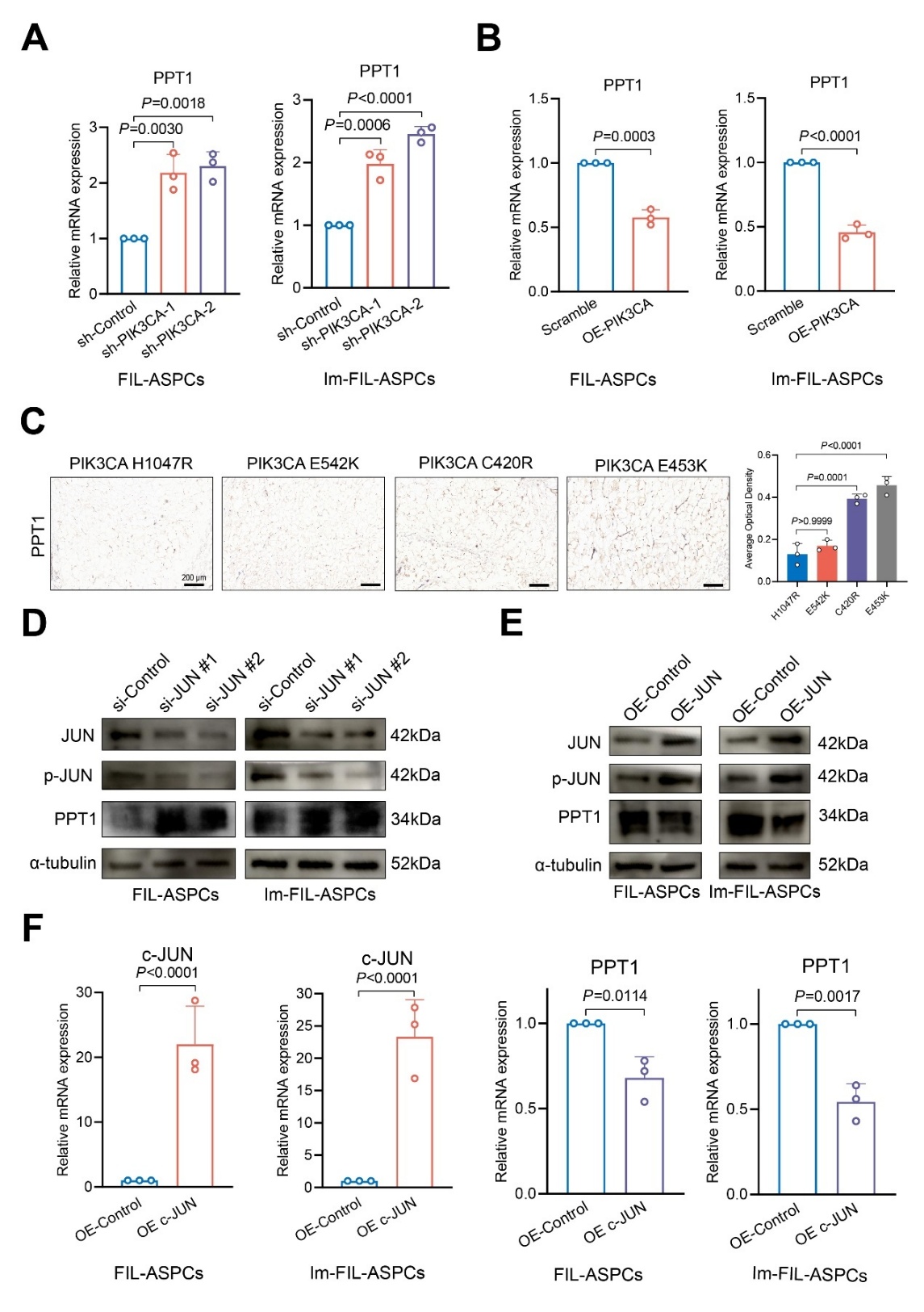


**Figure S3: c-JUN suppressed PPT1 expression.** A: RT-qPCR results showed PPT1 mRNA level in FIL-ASPCs and Im FIL-ASPCs after PIK3CA knockdown. B: RT-qPCR results showed PPT1 mRNA level in FIL-ASPCs and Im FIL-ASPCs after PIK3CA overexpression. C: Immunohistochemical staining revealed PPT1 expression levels in adipose tissues harboring distinct PIK3CA mutation sites. D: Western blot analysis showing PPT1 expression levels in FIL-ASPCs and Im FIL-ASPCs after c-JUN knockdown. E: Western blot analysis showing PPT1 expression levels in FIL-ASPCs and Im FIL-ASPCs after c-JUN overexpression. F: RT-qPCR results showed c-JUN and PPT1 mRNA level in FIL-ASPCs and Im FIL-ASPCs after c-JUN overexpression. Experiments were independently replicated at least three times with similar results (biological replicates). Data were analyzed by unpaired two-sided Student’s t tests (B and F) or one-way ANOVA (A and C) and were presented as mean ± SD with three replicate experiments.


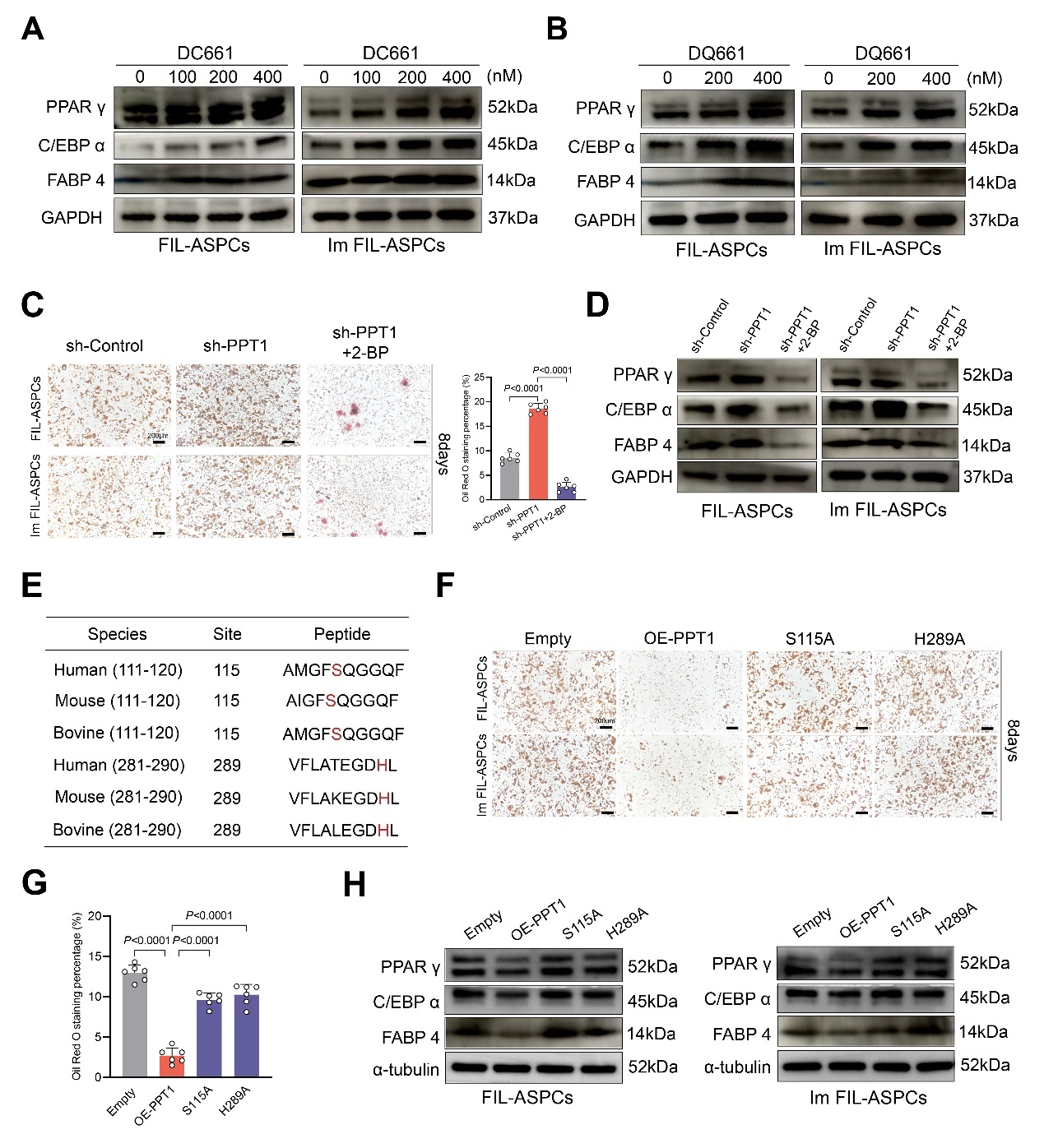


**Figure S4: PPT1 suppressed adipogenic differentiation via its depalmitoylation activity.** A: Western blot analysis showed the expression of PPAR γ, C/EBP α, FABP 4 in FIL-ASPCs and Im FIL-ASPCs treated with DC661. B: Western blot analysis showed the expression of PPAR γ, C/EBP α, FABP 4 in FIL-ASPCs and Im FIL-ASPCs treated with DQ661. C: Oil red O staining showed the lipid accumulation in PPT1-knockdown FIL-ASPCs and Im FIL-ASPCs after 2-BP treatment. D: Western blot analysis showed the expression of PPAR γ, C/EBP α, FABP 4 in PPT1-knockdown FIL-ASPCs and Im FIL-ASPCs after 2-BP treatment. E: Schematic alignment of amino-acid sequences flanking PPT1 S115 and H289 across different species. F: Oil red O staining showed the lipid accumulation in FIL-ASPCs and Im FIL-ASPCs transfected with PPT1 WT/S115A/H289A. G: Quantification of Oil red O staining area in FIL-ASPCs and Im FIL-ASPCs transfected with PPT1 WT/S115A/H289A. H: Western blot analysis showed the expression of PPAR γ, C/EBP α, FABP 4 in FIL-ASPCs and Im FIL-ASPCs transfected with PPT1 WT/S115A/H289A. Experiments were independently replicated at least three times with similar results (biological replicates). Data were analyzed by one-way ANOVA (C and G) and were presented as mean ± SD with three replicate experiments.


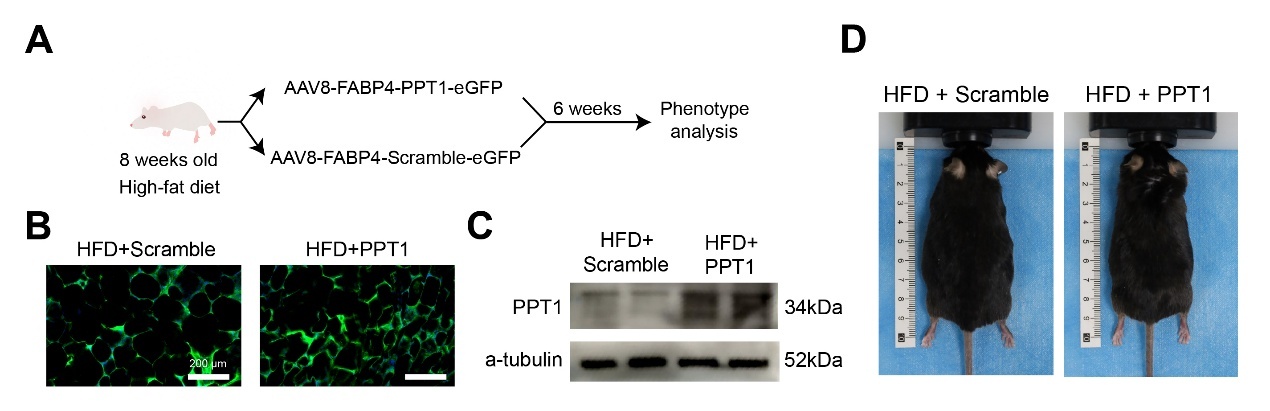


**Figure S5: AAV-mediated PPT1 adipocytic overexpression alleviated adipose hyperplasia induced by high-fat diet.** A: Schematic diagram of the experimental process in mice (n=6 per group). B: Representative image of eGFP expression 6 weeks post injection of AAV. Scale bar: 200μm. C: PPT1 expression was analyzed by western blot in adipose tissue of HFD-PPT1 group and HFD-Scramble group. D: Overview of the body size of the mice from different groups.


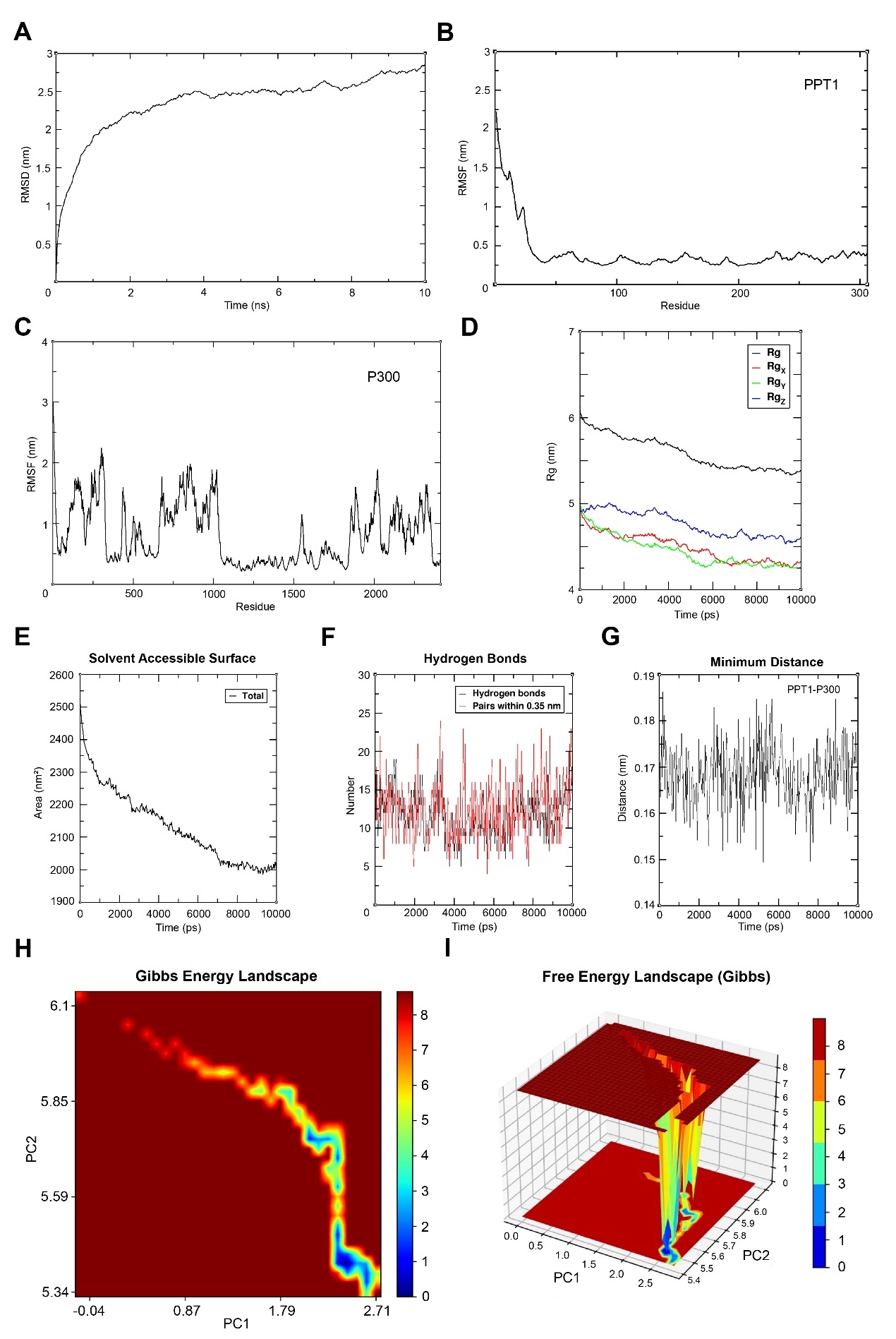


**Figure S6: Molecular dynamics simulation analysis of the PPT1-P300 complex.** A: The RMSD-versus-time curve reflects the overall stability of the protein-protein complex during the 10 ns molecular dynamics simulation. B: The RMSF plot depicts the flexibility changes of the PPT1 protein during the molecular dynamics simulation. C: The RMSF-versus-residue curve illustrates the residue-specific flexibility profile of P300 throughout the molecular dynamics simulation. D: Evolution of the radius of gyration (Rg) for the system during the 0-10 ns molecular dynamics trajectory (time in ps). E: Variation of the solvent-accessible surface area (SASA) of the system throughout the 10-ns molecular dynamics simulation. F: Number of interfacial hydrogen bonds (black line) and donor-acceptor pairs within 0.35 nm (red line) during the 10-ns simulation. G: Time evolution of the minimum inter-atomic distance between PPT1 and P300 during the molecular dynamics simulation. H: Free-energy landscape (FEL) plotted with RMSD (conformational deviation) on the x-axis and Rg (radius of gyration, reflecting overall compactness) on the y-axis; colors encoded Gibbs free energy, with blue indicating low-energy (most stable) states and red high-energy (unstable) states. I: Three-dimensional free-energy landscape (Gibbs energy) derived from molecular-dynamics simulations. The horizontal axis (PC1) denoted RMSD, reporting conformational deviation; the vertical axis (PC2) corresponded to Rg, reflecting overall compactness; the height axis represented Gibbs free energy. Color and elevation jointly encode energy magnitude: blue depressions marked the lowest-energy, most stable conformations, whereas red plateaus indicated high-energy, unstable regions.


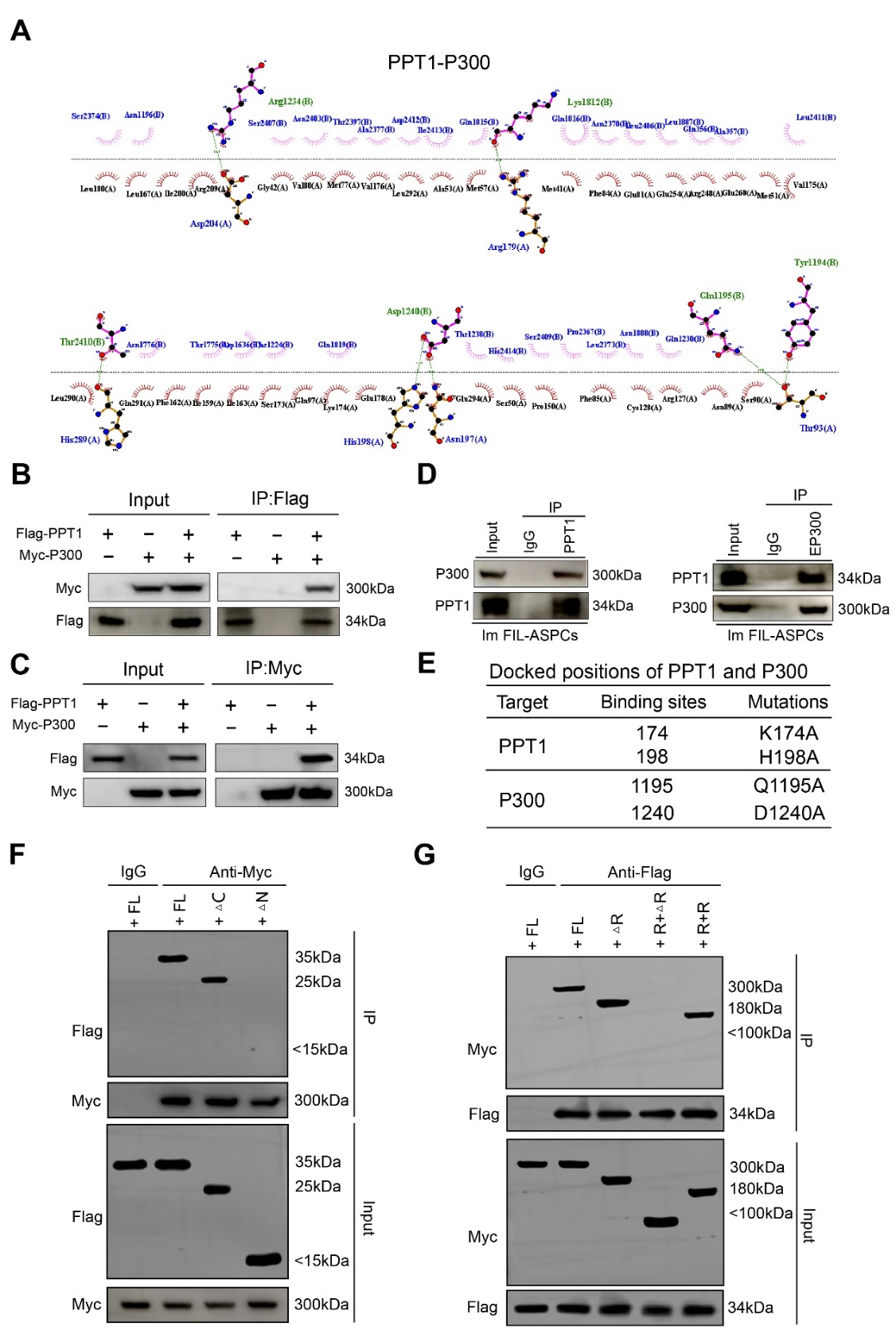


**Figure S7: The physical interaction between PPT1 and P300.** A: Detailed hydrogen bonding sites between amino acids in PPT1 and P300. B-C: The exogenous interaction between PPT1 and P300 was detected by Co-IP and western blotting assays. D: Im FIL-ASPCs cellular lysates were analyzed by Co-IP followed by western blotting. E: Docked positions of PPT1 and P300 predicted by PDBePISA and design of the mutations of binding sites between PPT1 and P300. F: IP and western blot assay indicating the interactions between FLAG-tagged truncated PPT1 and Myc-tagged P300 proteins in HEK293T cells. Cell extracts were IP with an anti-Myc antibody. G: IP and western blot assay indicating the interactions between FLAG-tagged PJA2 and Myc-tagged truncated P300 proteins in HEK293T cells. Cell extracts were IP with an anti-Flag antibody.


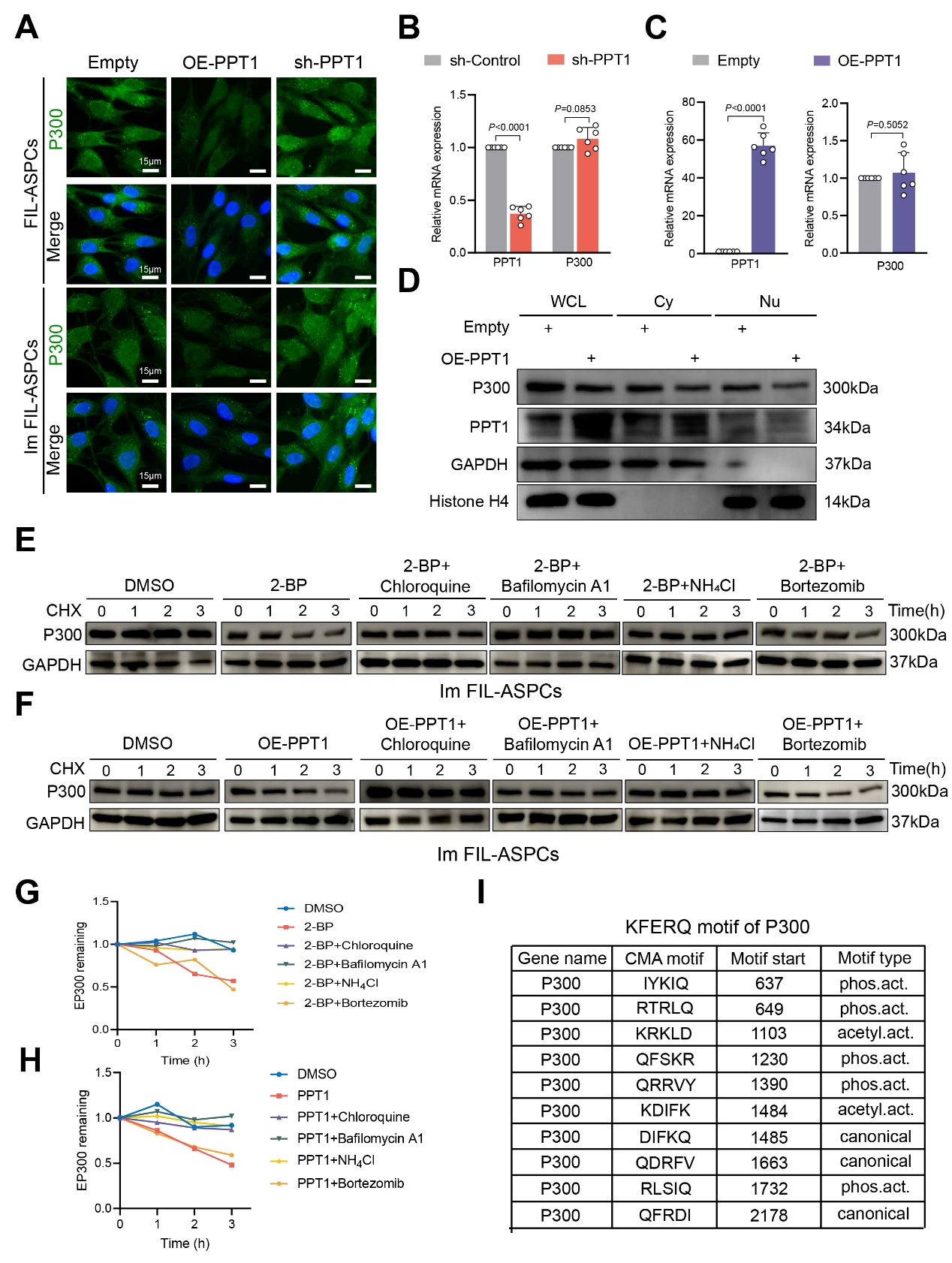


**Figure S8: PPT1 promoted the lysosome-dependent degradation of P300.** A: Immunofluorescence staining showed the cytoplasm/nucleus distribution of P300 in FIL-ASPCs and Im FIL-ASPCs with or without PPT1 interference. Scale bar: 15 μm. B: RT-qPCR analysis for the mRNA expression level of PPT1 and P300 in Im FIL-ASPCs transfected with PPT1 shRNAs. C: RT-qPCR analysis for the mRNA expression level of PPT1 and P300 in PPT1-overexpression Im FIL-ASPCs. D: Nucleus/cytoplasmic P300 quantification of Im FIL-ASPCs with or without PPT1 overexpression. E: Im FIL-ASPCs were subjected to CHX-chase analysis in the absence or presence of the 2-BP, combined with lysosome-blocking agents (chloroquine, bafilomycin A1 or NH₄Cl) or the proteasome inhibitor bortezomib. F: Im FIL-ASPCs with or without PPT1 overexpression were subjected to CHX-chase analysis, combined with lysosome-blocking agents (chloroquine, bafilomycin A1 or NH₄Cl) or the proteasome inhibitor bortezomib. G: Quantification of the intensity measured by the relative level of P300 remaining in (E). H: Quantification of the intensity measured by the relative level of P300 remaining in (F). I: Detailed information of KFERQ-like motifs in human P300. Data were analyzed by unpaired two-sided Student’s t tests (B and C) and were presented as mean ± SD with three replicate experiments.


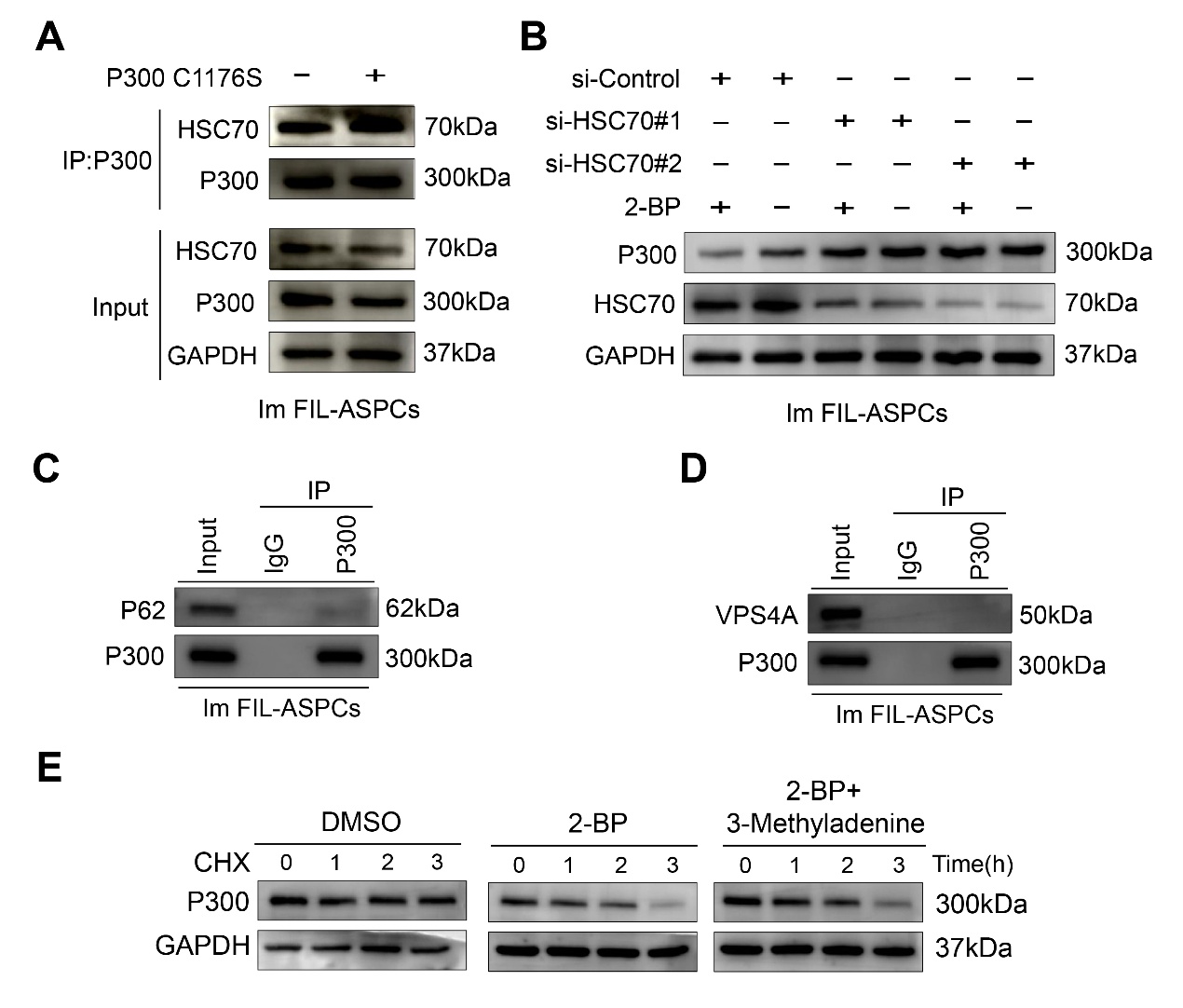


**Figure S9: P300 palmitoylation suppressed the chaperone-mediated autophagy.** A: Co-IP assays were performed to determine whether P300 interacted with HSC70 in Im FIL-ASPCs transfected with P300 C1176S. B: Western blot analysis of Im FIL-ASPCs transfected with HSC70 siRNAs after 2-BP treatment. C-D: The exogenous interaction between P62 or VPS4A and P300 was detected by Co-IP and western blotting assays. E: Im FIL-ASPCs were subjected to CHX-chase analysis in the absence or presence of the 2-BP, combined with macroautophage-blocking agents.


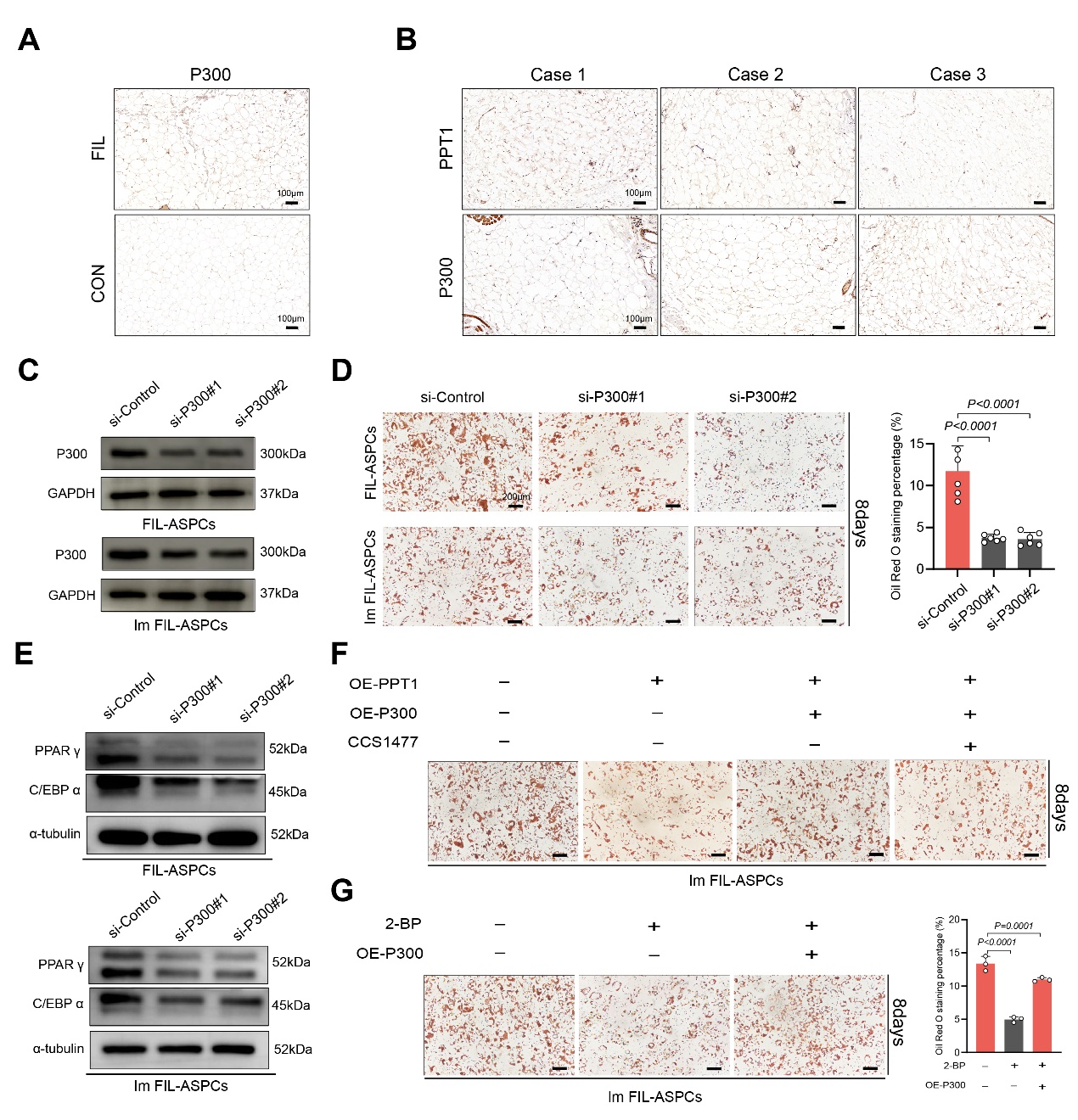


**Figure S10: PPT1 inhibited adipogenesis through P300.** A: Immunohistochemical staining revealed the expression levels of P300 in FIL and control adipose tissues. B: Immunohistochemical staining revealed the expression levels of P300 and PPT1 in FIL adipose tissue. C: Western blot analysis showed the P300 expression in FIL-ASPCs and Im FIL-ASPCs transfected with P300 siRNA. D: Oil red O staining presented the lipid accumulation in FIL-ASPCs and Im FIL-ASPCs transfected with P300 siRNA after adipogenic induction for 8 days. E: Western blot analysis showed the PPAR γ and C/EBP α expression in FIL-ASPCs and Im FIL-ASPCs transfected with P300 siRNA after adipogenic induction for 3 days. F: Oil red O staining presented the lipid accumulation in Im FIL-ASPCs transfected with WT PPT1/P300. G: Oil red O staining presented the effect of 2-BP in lipid accumulation in PPT1-overexpression Im FIL-ASPCs. Data were analyzed by one-way ANOVA (D and G) and were presented as mean ± SD.


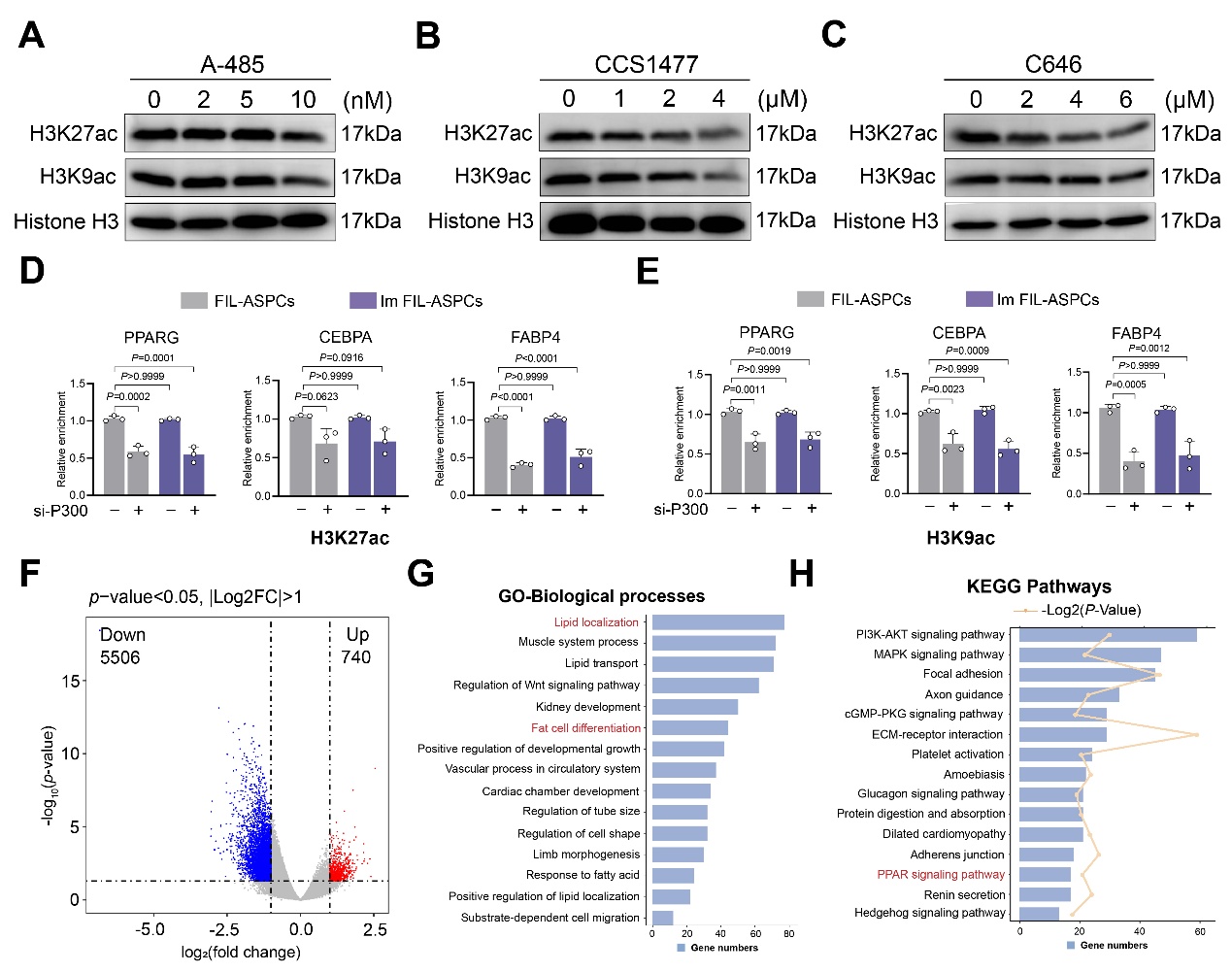


**Figure S11:** **P300 regulated chromatin opening.** A: Western blot analysis showed the H3K27ac and H3K9ac expression in Im FIL-ASPCs treated with different concentrations of A-485. B: Western blot analysis showed the H3K27ac and H3K9ac expression in Im FIL-ASPCs treated with different concentrations of CCS1477. C: Western blot analysis showed the H3K27ac and H3K9ac expression in Im FIL-ASPCs treated with different concentrations of C646. D: H3K27ac ChIP-qPCR validation for selected gene promoter regions (PPARG, CEBPA, FABP4) in FIL-ASPCs and Im FIL-ASPCs with different interventions. E: H3K9ac ChIP-qPCR validation for selected gene promoter regions (PPARG, CEBPA, FABP4) in FIL-ASPCs and Im FIL-ASPCs with different interventions. F: Volcano map showing the differentially accessible regions (DARs) in FIL-ASPC after P300 knockdown detected by ATAC-seq. G: GO analysis of the DARs peaks at candidate target genes. H: KEGG analysis of the DARs peaks at candidate target genes.
