# Supplementary material for: PIK3CA Mutations Downregulate PPT1 to Promote Adipogenesis by Suppressing P300 Depalmitoylation and Phase Separation
Source: Adv Sci (Weinh). 2026 Jan 29;13(19):e23139. doi: 10.1002/advs.202523139 (PMC13045223; doi:10.1002/advs.202523139)
Supplement: Supplementary file 2 — Supplemental File 2: advs74036‐0002‐Tables.zip. [file ADVS-13-e23139-s001.zip › Table S3.docx]

| **Table S3. Prime sequence for shRNA and siRNA** | |
| --- | --- |
| Genes | Primer information (5'→3') |

sh-Control CCTAAGGTTAAGTCGCCCTCG

sh-PIK3CA-1 GCTTGAAGAGTGTCGAATTAT

sh-PIK3CA-2 AGAATATCAGGGCAAGTATAT

sh-PPT1-1 CCCATAAAGGAGGATGTGTAT

sh-PPT1-2 CCTGTAGATTCGGAGTGGTTT

SiRNA sequence

| Genes | Primer information (5'→3') |
| --- | --- |

Control-siRNA UUCUCCGAACGUGUCACGUTT

P300-siRNA1 CGACUUACCAGAUGAAUUATT

P300-siRNA2 GCACAAAUGUCUAGUUCUUTT

c-JUN-siRNA1 CCAAGAACGUGACAGAUGATT

c-JUN-siRNA2 AGAUGGAAACGACCUUCUATT

HSC70-siRNA1 CGUCUGAUUGGACGCAGAUUUTT

HSC70-siRNA2 CCAAGACUUCUUCAAUGGAAATT
