# Supplementary material for: PIK3CA Mutations Downregulate PPT1 to Promote Adipogenesis by Suppressing P300 Depalmitoylation and Phase Separation
Source: Adv Sci (Weinh). 2026 Jan 29;13(19):e23139. doi: 10.1002/advs.202523139 (PMC13045223; doi:10.1002/advs.202523139)
Supplement: Supplementary file 2 — Supplemental File 2: advs74036‐0002‐Tables.zip. [file ADVS-13-e23139-s001.zip › Table S2.docx]

**Table S2. Primers used in this study**

**Primer for qPCR:**

| **Gene/RNA** | **Forward primer (5'→3')** | **Reverse primer (5'→3')** |
| --- | --- | --- |
| GAPDH | CATCATCCCTGCCTCTACTGG | GTGGGTGTCGCTGTTGAAGTC |
| PIK3CA | TCGCCTCATAGCAGAGCAAT | AGGACAACAACATGCTCCGA |
| PPT1 | AGGACGTGGAGAACAGCTTC | GAGCCACTGCCCTCAGAAAT |
| P300 | TCCGAGACATCTTGAGACGACAG | GGGTTGCTGGAACTGGTTATGG |
| ZDHHC12 | ACCCTGGCTACGTGAATGTG | TGCAGCACCAGGCAGTATC |
| c-JUN | CCAACTCATGCTAACGCAGC | TCTCTCCGTCGCAACTTGTC |
|  |  |  |

**Primer for ChIP-qPCR**

| **Gene/RNA** | **Forward primer (5'→3')** | **Reverse primer (5'→3')** |
| --- | --- | --- |
| PPARG | TCCTGGGAGCCTAACTGAG | CTTCCACCAAGGGACCTGAG |
| CEBPA | CTCCTCCTGCCTGCCCTA | GTGCAGCCTCGGGATACTC |
| FABP4 | TGGTTCTCCCTGGCAAATAG | CATTAAGCTGTCAAAACAGGAATG |
